# Two-dimensional heterostructures built from ultrathin CeO_2_ nanosheet surface-coordinated and confined metal–organic frameworks with enhanced stability and catalytic performance†

**DOI:** 10.1039/d2sc00308b

**Published:** 2022-02-14

**Authors:** Haiyan An, Yang Hu, Nan Song, Tingliang Mu, Shiqiang Bai, Yong Peng, Liangliang Liu, Yu Tang

**Affiliations:** State Key Laboratory of Applied Organic Chemistry, Key Laboratory of Nonferrous Metal Chemistry and Resources Utilization of Gansu Province, College of Chemistry and Chemical Engineering, Lanzhou University Lanzhou 730000 China liull@lzu.edu.cn tangyu@lzu.edu.cn; Key Laboratory of Magnetism and Magnetic Materials of Ministry of Education, School of Physical Science and Technology, Lanzhou University Lanzhou 730000 China

## Abstract

Two-dimensional (2D) metal–organic framework (MOF) based heterostructures will be greatly advantageous to enhance catalytic performance because they increase the contact surface and charge transfer. Herein, a novel 2D heterostructure named CeO_2_@NiFe-MOFs, in which monolayer NiFe-MOFs is coordinated with ceria (CeO_2_) to improve catalytic and stability performance, is successfully constructed by the strategy of *in situ* growth on the surface of ultrathin CeO_2_ nanosheets being functionalized with monolayer carboxylic acid groups. The 2D heterostructure possesses a sandwich structure, where monolayer NiFe-MOFs are coordinated to both the top and bottom surface of CeO_2_ nanosheets *via* joining carboxylic acid groups. In particular, CeO_2_ with robust coordination plays a significant role in the anchoring of carboxylic acid groups and binding strength of heterostructures. The 2D CeO_2_@NiFe-MOF heterostructure with a joint effect of metal–ligand coordination not only presents good structural stability but also significantly enhances the oxygen evolution reaction (OER) efficiencies in comparison to bare NiFe-MOFs, achieving a current density of 20 mA cm^−2^ at a low overpotential of 248 mV as well as durability for at least 40 h. Meanwhile, the electronics, optics, band gap energy and local strains of CeO_2_ decorated with 2D NiFe-MOFs are different to the properties of bare CeO_2_. Our study on the construction of an ultrathin CeO_2_ surface-coordinated and confined MOF layer may pave a new way for novel 2D MOF composites/heterostructures or multi-functional 2D CeO_2_ materials to be used in energy conversion or other fields.

## Abstract 2

A synthetic strategy to prepare 2D heterostructures from ultrathin CeO_2_ surface-coordinated metal–organic framework was proposed, proving multiple effects of metal-coordinated interactions in 2D heterostructures.

## Graphical abstract

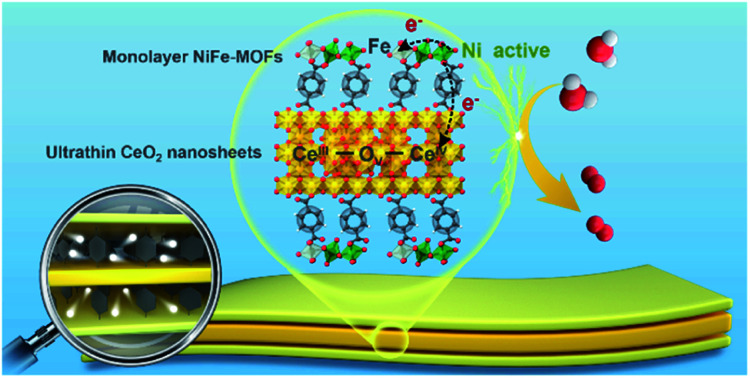

## Introduction

The energy crisis and environmental pollution are the most crucial challenges facing humanity today. Electrocatalytic water splitting plays a key role in addressing issues of energy sustainability and environmental protection.^1^ The development of a high-performance electrocatalyst for the oxygen evolution reaction (OER) with sluggish kinetics is a great challenge in the field. Heterostructures have attracted great attention in the fields of materials, catalysis and energy, because the unique structures generally exhibit enhanced catalytic activity in comparison with other uniformly structured counterparts.^2,3^ Metal–organic frameworks (MOFs) are a class of porous crystal coordination polymers, which are commonly constructed by the coordination of metal nodes with diversiform organic ligands,^4^ possessing ultrahigh porosity, enormous surface areas, accessible metal active sites, hybrid composition, good designability and structural diversity. Benefitting from these merits, MOFs have attracted considerable interest in electrocatalytic water splitting.^5^ However, many bulk MOFs still suffer from poor conductivity, buried active sites and chemical instability caused by the intrinsic shortages of organic ligands. Compared to bulk MOFs, ultrathin two-dimensional (2D) MOFs afford not only an enlarged surface area but also more active sites exposed on the surface, which can be beneficial to the adsorption and activation of reactants and improvement of catalytic performance.^6^ MOF-based heterostructures can concurrently provide architectural features of MOFs and additional physicochemical properties.^7,8^ Therefore, to take full advantage of MOFs, ultrathin 2D MOFs and MOF-based heterostructures as efficient electrocatalysts in the field of water splitting and related energy conversion have aroused the attention of researchers.

Ultrathin 2D MOFs with thickness on a nanometer scale are beneficial to improving the performance of electrocatalytic water splitting.^9,10^ Accordingly, two fabrication strategies have been developed to synthesize ultrathin 2D MOFs, including the top-down strategy (exfoliation methods) and bottom-up strategy (direct syntheses from metal and organic precursors).^11^ The exfoliation method mainly assisted by ultrasound is only applicable to bulk MOFs with intralayer coordinated bonds that can be easily broken, resulting in inhomogeneity, fragmentation and restacking of MOF-nanosheets.^12^ On the other hand, the bottom-up strategy has proved to be an efficient method to prepare ultrathin 2D MOFs. For example, the ultrathin 2D Co-MOF nanosheets (thickness of ∼2 nm), prepared under hydrothermal conditions using polyvinylpyrrolidone as the surfactant, showed high intrinsic activity and enhanced electrical conductivity.^13^ The surfactant may block some of the active sites. In order to avoid the introduction of an organic surfactant, recently, Lang and co-workers prepared ultrathin Ni–M-MOFs (M = Fe, Al, Co, Mn, Zn, and Cd) with a thickness of several atomic layers *via* a one-step hydrothermal method, where the solvent played a crucial role in modulating the formation of 2D MOFs.^14^ Although many research achievements have been achieved in the preparation of 2D MOFs, developing the ideal technology to simultaneously control the growth of 2D MOFs along the lateral direction only and suppress vertical growth to the nanometer scale still remains challenging.^15^ 2D MOF-based heterostructures can provide the architectural features of nanometer MOFs; moreover, heterostructures can achieve the controllable growth of ultrathin MOFs. Therefore, the novel functional heterostructures, especially those fabricated from 2D MOFs or other 2D materials, have become the current focus of research on electrocatalytic water splitting, due to their increased contact surface and enhanced charge transfer.^16,17^

Various strategies have been reported to fabricate MOF-based heterostructures, such as spin coating,^18^ electrochemical deposition,^19^ layer-by-layer assembly (LBL),^20^*in situ* growth^21^ and so on.^22^ However, most of the proposed strategies are not suitable to uniquely construct heterostructures by the controllable integration of 2D MOFs and nanomaterials with ultrathin thickness and efficient activities. Therefore, it is urgent to develop a facile and efficient method to synthesize heterostructures of 2D MOFs with 2D nanomaterials. Moreover, the accurate selection of 2D nanomaterials with good properties to support and control the growth of MOFs will lend a hand to improve the activities and stabilities of the heterostructures. Among the abundant 2D nanomaterials,^23^ ceria (CeO_2_) is widely used to improve the performance of materials in catalytic conversion processes.^24^ Its excellent stability and corrosion resistance also result in the structural robustness and durability of materials.^25^ In particular, ultrathin CeO_2_ nanosheets display the largest specific surface area, coordination-unsaturated Ce sites and a surface defect-rich structure, which is conducive to catalytic performance.^26^ As far as we are concerned, CeO_2_ is an ideal substrate for coordinated functionalization of carboxylic acid groups, which provides a strategy for the design of new CeO_2_-based materials with tailored multiple functionalities. Considering all the above, we envisage that ultrathin CeO_2_ would be an ideal nanomaterial to support MOFs. Besides, a Ce-decorated transition metal was reported by Zheng and co-workers,^27^ and to the best of our knowledge, the heterostructures of ultrathin CeO_2_ nanosheets with surface-coordinated and confined transition metal-MOFs were poorly understood until fairly recently.

Herein, we report the coordinated functionalization of ultrathin CeO_2_ nanosheets by attaching oxygen-containing carboxylic functional groups to their surface *via* an approach of ligand exchange, and then using a straightforward protocol of the coordination between the carboxylic groups and the metal ions to form monolayer MOFs on the surface of CeO_2_ and construct CeO_2_@NiFe-MOFs heterostructures. Since the surface of CeO_2_ can expose coordination-unsaturated Ce atoms, which is beneficial to coordinate with oxygen-containing functional groups, ligand exchange has been adopted to anchor the oxygen-containing functional groups. We demonstrate that the protocol is not only beneficial for the chemical functionalization of CeO_2_ with tailored oxygen-containing functional groups but also affords well-defined 2D CeO_2_@NiFe-MOF heterostructures, in which ultrathin CeO_2_ nanosheets with the largest specific surface area provide a substrate for the dispersion of the 2D NiFe-MOF layer and the thickness of the 2D NiFe-MOFs could be controlled down to nanometers. Therefore, ultrathin 2D heterostructures efficiently increase the contact surface to capture the targeted molecules and will accelerate charge transfer between active NiFe-MOFs and inorganic CeO_2_. In addition, compared with the properties of bare CeO_2_, CeO_2_ decorated with 2D NiFe-MOFs exhibits different properties of electronics, optics, band gap energy and local strain. Meanwhile, 2D CeO_2_@NiFe-MOF heterostructures assisted by CeO_2_ nanosheets can be used as an electrocatalyst, exhibiting significantly enhanced performance and long-term stability in the OER.

## Results and discussion

**Fig. 1:**
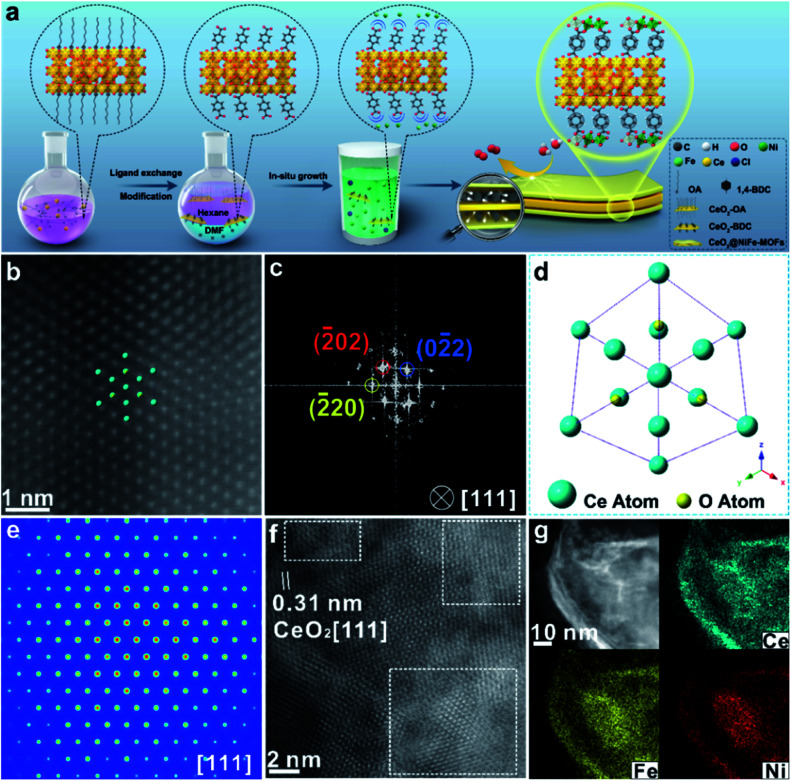
(a) Schematic illustration of the CeO_2_@NiFe-MOF heterostructure preparation procedure including two steps: two-phase ligand exchange and *in situ* growth. (b) Atomic HAADF-STEM image of CeO_2_@NiFe-MOFs along the [111] orientation of CeO_2_. (c) Corresponding FFT pattern from (b). (d) The projected structural model of CeO_2_ in CeO_2_@NiFe-MOFs. (e) FFT-filtered atomic resolution simulation image. (f) Enlarged HR-TEM image of CeO_2_@NiFe-MOFs from Fig. S10e.† (g) The corresponding elemental mapping images of CeO_2_@NiFe-MOF heterostructures.

Ultrathin heterostructures have been prepared through an *in situ* growth strategy on the CeO_2_ surface functionalized by carboxylic acid groups. As shown in Fig. 1a, the ultrathin 2D CeO_2_@NiFe-MOF heterostructures are prepared *via* a two-step procedure (ligand exchange and *in situ* growth). Firstly, terephthalic acid (1,4-BDC) coordinates with the cerium cation on the CeO_2_ surface, in place of oleic acid (OA) on the surface by two-phase ligand exchange. Thus, ultrathin CeO_2_ nanosheets were functionalized by monolayer carboxylic acid groups (CeO_2_-BDC).^28^ Secondly, CeO_2_-BDC provides the templates for the crystallization of MOFs, where monolayer NiFe-MOFs are coordinated to both the top and bottom surface of CeO_2_ nanosheets *via* joining carboxylic acid groups, obtaining ultrathin 2D CeO_2_@NiFe-MOF heterostructures with a sandwich structure. In the process of reaction, when CeO_2_-BDC is suspended in an alkaline solution, the carboxylic acid groups can be polarized in the alkaline solution, and then uniformly adsorb metal ions (*e.g.* Ni^2+^, Fe^2+^) *via* strong electrostatic adsorption. As a consequence, MOFs crystallize onto the CeO_2_ surface as films. Undoubtedly, these 2D templated 1,4-BDC tails coordinated with metal ions can create abundant open metal sites, and the 1,4-BDC bridged heterostructures featured 2D layered structures, facilitating the increased interlaminar porosity and largely promoted ion transport.

CeO_2_-OA nanosheets with an ultrathin thickness and organic ligand coordinately functionalized ultrathin CeO_2_ were prepared according to previous reports (Fig. S1–S3†).^29,30^ The success of coordinated modification of organic ligands on CeO_2_ nanosheets can be observed by vibrational spectroscopy (Fig. S4 and S5†).^31^ In this work, we mainly focus on CeO_2_-BDC nanosheets. CeO_2_-OA and CeO_2_-BDC were investigated by powder X-ray diffraction (PXRD). As shown in Fig. S6,† the shifted diffraction peaks of CeO_2_-BDC compared to that of pristine CeO_2_-OA can probably be ascribed to the partial transition from Ce^3+^ to Ce^4+^ in the process of substitution. The appearance of a peak at ≈15.38° can be ascribed to the coordinated combination of the Ce-ligand.^32^ The water contact angle was also measured to further investigate the CeO_2_ surface (Fig. S7†). The above results elucidate the successful functionalization of carboxylic acid groups onto the CeO_2_ surface.

The transmission electron microscopy (TEM) image of CeO_2_-OA shows the characteristic structure of nanosheets with diverse lateral sizes (Fig. S8a†). The nanosheet structure is well preserved in CeO_2_-BDC nanosheets (Fig. S8b†) with a relatively more disrupted morphology compared to pristine CeO_2_-OA nanosheets. Morphologies of CeO_2_-OH and CeO_2_-PMA (Fig. S9†) are also strikingly different from CeO_2_-OA, although nanosheets can be observed. The dramatic change in the morphologies of CeO_2_-BDC, CeO_2_-OH and CeO_2_-PMA is mainly caused by coordination modification, which can be regarded as a sign of chemical modification. The TEM images of 2D CeO_2_@NiFe-MOF heterostructures (Fig. S10a and b†) show an ultrathin sandwich-like structure with multiple stacking faults. High-resolution TEM (HR-TEM) reveals the crystal structure of 2D CeO_2_@NiFe-MOF heterostructures. As depicted in Fig. S10c,† the lattice spacing of CeO_2_ is measured to be 0.31 nm corresponding to the [111] interplanar spacing, and the *d* = 0.21 nm corresponds to the interplanar distance in the [103] plane of NiFe-MOFs.^14,33^ The corresponding selected area electron diffraction (SAED) pattern (Fig. S10d†) can be indexed to the [111] and [103] lattice planes of CeO_2_ and NiFe-MOFs, respectively. The high-angle annular dark field aberration corrected scanning TEM (HAADF-STEM) image reveals the fine structure of CeO_2_ within CeO_2_@NiFe-MOF heterostructures (Fig. 1b). The fast Fourier transform (FFT) image in Fig. 1c shows the coexistence of two clearly distinct structural domains. Fig. 1d shows the corresponding crystal structure of CeO_2_ unit cells in CeO_2_@NiFe-MOF heterostructures taken along the [111] orientation. The FFT-filtered atomic resolution image was further used to visualize the corroboration along the [111] orientation (Fig. 1e), and Fig. S11† shows the corresponding three-dimensional atom intensity profile. 2D NiFe-MOFs are nonuniform on the surface of CeO_2_ nanosheets, and this may be attributed to the nonuniformity of the monolayer carboxylic acid groups (Fig. 1f). The elemental composition of CeO_2_@NiFe-MOF heterostructures is identified by HAADF-STEM mapping (Fig. 1g and S10f†), confirming the uniform distribution of Ce, Ni, Fe and O elements throughout the entire nanosheets.

**Fig. 2:**
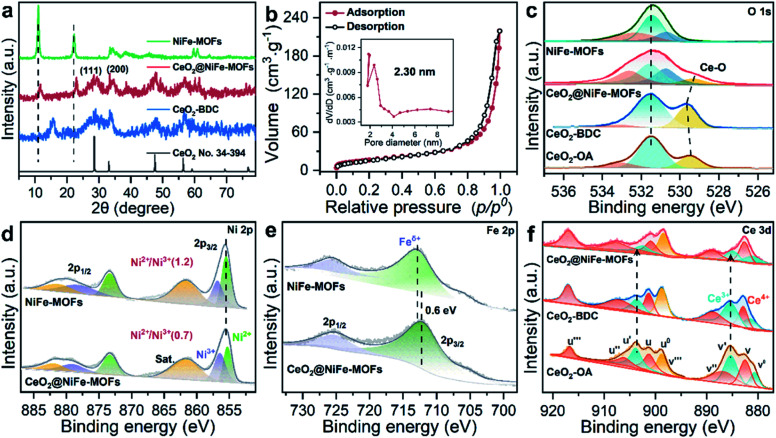
(a) PXRD patterns of CeO_2_ (no. 34-394), CeO_2_-BDC, CeO_2_@NiFe-MOFs and NiFe-MOFs. (b) Nitrogen adsorption/desorption isotherm of CeO_2_@NiFe-MOFs, pore size distributions (inset). (c–f) XPS spectra of O 1s, Ni 2p, Fe 2p and Ce 3d; Ce 3d XPS spectra reveal the change of valence of Ce caused by coordinated functionalization.

The structure of CeO_2_@NiFe-MOF heterostructures is further confirmed by the PXRD pattern (Fig. 2a), in which the decrease of crystallinity results from the increased amounts of open metal sites.^34^ The diffraction peaks of CeO_2_@NiFe-MOF heterostructures are slightly shifted, revealing structural transformation induced by ligand distortion. Comparing Raman spectra of CeO_2_@NiFe-MOF heterostructures with those of NiFe-MOFs, the intensity of CeO_2_@NiFe-MOF heterostructures at 525 cm^−1^ is found to be considerably lower (Fig. S12†). The atomic force microscopy (AFM) tests are further performed to observe the thickness and surface of CeO_2_-OA and CeO_2_@NiFe-MOFs deposited on silicon substrates. As shown in Fig. S13,† compared to CeO_2_-OA, CeO_2_@NiFe-MOF heterostructures exhibit a significant change of thickness in the range of 3.8–8 nm and surface morphology. These results verified the successful synthesis of ultrathin CeO_2_@NiFe-MOF heterostructures. Fig. 2b shows the specific surface area of CeO_2_@NiFe-MOFs which is estimated to be 53 m^2^ g^−1^. Besides, the pore size distribution can also be seen from the inset of Fig. 2b, exhibiting a pore diameter of 2.3 nm, allowing the faster diffusion of ions. The thermogravimetric (TG) analysis also demonstrated good stability of NiFe-MOF nanosheets in CeO_2_@NiFe-MOFs (Fig. S14†).

Surface-sensitive X-ray photoelectron spectroscopy (XPS) characterization was carried out to establish the surface elemental composition and valence state, which helps to analyze the intrinsic electronic structure of the prepared materials. As shown in the full XPS spectra of CeO_2_@NiFe-MOF heterostructures (Fig. S15†), Ce, Ni, Fe, O, and C elements were identified. In the high-resolution O 1s XPS spectra (Fig. 2c), the broad peak at 529 eV can be assigned to the characteristic peak of CeO_2_;^25,35,36^ the O 1s of CeO_2_-BDC (529.6 eV) shifts to higher binding energy compared to that of CeO_2_-OA (529.3 eV). A much broader peak at around 529.3 eV in CeO_2_@NiFe-MOFs suggests possible electronic interactions between carboxylic acid groups and CeO_2_. The ratio of the Ni^II^/Ni^III^ XPS peak in Ni (2p_3/2_) is used as the reference, and the Ni^II^/Ni^III^ ratio on the surface of CeO_2_@NiFe-MOFs (0.7) decreases compared with the NiFe-MOFs (1.2) (Fig. 2d), suggesting that the Ni^II^ is oxidized when NiFe-MOFs is coordinated with CeO_2_. The Fe peaks of CeO_2_@NiFe-MOFs show apparent shifts toward the lower binding energies (Fig. 2e). The Fe^3+^ sites serve as Lewis acid sites,^37^ and Ce atoms act as electron-accepting sites, confirming partial electron transfer from Ni to Fe and Ce atoms.^38^

XPS spectra of Ce 3d are shown in Fig. 2f, the peaks v′′′, v′′, v′ and v^0^ arise from the ionization of the 3d_5/2_ electron, the u′′′, u′′, u′, u and u^0^ peaks arise from the 3d_3/2_ electron, and the peaks (labeled v^0^, v′, u^0^, u′) are coincident with previously reported XPS peaks of Ce^3+^ of the Ce_2_O_3_ compound.^39^ It is clear that CeO_2_-OA shows binding energy related to Ce^3+^, while CeO_2_-BDC shows decrease of intensity of Ce^3+^ related peaks, indicating the efficient replacement of OA by 1,4-BDC, which leads to a partial transition from Ce^3+^ to Ce^4+^. For CeO_2_@NiFe-MOFs, the Ce^3+^ related peaks at 903.8 and 885.5 eV dramatically decrease, showing features related to Ce^4+^. The comparison of results shows that coordinated functionalization to CeO_2_-OA may induce gradual oxidation of Ce^3+^. Therefore, the interactions between CeO_2_ and organic ligands can be testified according to the electronic charge transfer. To further confirm valence states of Ce ions in CeO_2_@NiFe-MOFs, the Ce-M edge was measured by STEM-EELS (electron energy loss spectroscopy), which was performed to directly collect the cerium signal. EELS spectra are depicted in Fig. S16,† where the intensity of the M_4_ edge is higher than that of the M_5_ edge, which is attributed to Ce^4+^.^40,41^ The result of EELS spectral analysis for the valence state of Ce ions is coincident with that of the XPS spectral analysis. The valence of Ce ions is nominally +4 in CeO_2_@NiFe-MOFs, indicating that reversible charge transfer is easier between the Ce^4+^ and Ce^3+^ oxidation stages, which is advantageous to the oxygen evolution reaction.

**Fig. 3:**
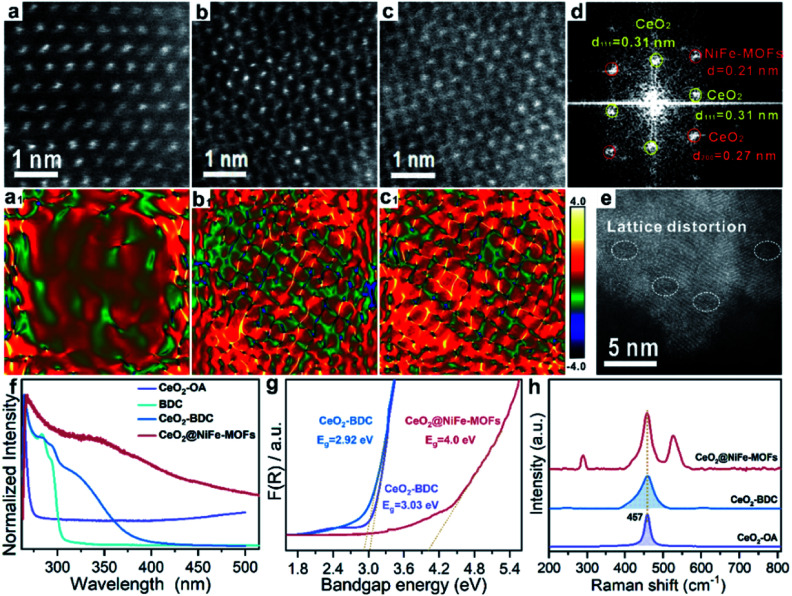
Characterization of lattice strain of CeO_2_@NiFe-MOFs. (a–c) The HR-TEM images of CeO_2_@NiFe-MOFs from the local magnification of Fig. S17.† (a_1_–c_1_) The corresponding strain mapping in (a–c). (d) FFT diffractogram of the HR-TEM lattice of (c). (e) HR-TEM image of CeO_2_@NiFe-MOFs. (f) UV-vis absorption spectra of CeO_2_-OA cyclohexane solution and CeO_2_-BDC, NiFe-MOFs and CeO_2_@NiFe-MOFs DMF solution. (g) The band gap energy of CeO_2_-OA, CeO_2_-BDC and CeO_2_@NiFe-MOFs calculated from the UV/Vis diffuse reflectance spectra. (h) The corresponding Raman spectroscopy.

The lattice distortion in CeO_2_ can be caused by first dissolution of surface Ce of CeO_2_ by carboxylic acid and then coordination of carboxylic acid groups onto the CeO_2_ surface. To further confirm the existence of lattice distortion and surface strains, the HR-TEM lattice images were used to measure the nanoscale spatial distribution of the strains (Fig. 3a–c). The degree of CeO_2_ surface strains is associated with the extent of dissolution of surface Ce in CeO_2_, and thus, surface strain is readily adjusted by organic ligands. CeO_2_ has a standard lattice spacing and thus ensures the degree of strain. Strain mapping measured by geometric phase analysis (GPA) can indicate the existence of legible CeO_2_ lattice strains upon coordinated modification, resulting from the interactions of CeO_2_ and organic ligands. The NiFe-MOFs on CeO_2_ nanosheets are negligible (Fig. 3a), giving CeO_2_ only slight lattice strain. As shown in Fig. 3b and c, the surface strain of CeO_2_ revert proportional to dense coverage of NiFe-MOFs, demonstrating that surface strain is caused by dissolution of surface Ce of CeO_2_. Meanwhile, the FFT diffractogram of the HR-TEM lattice in Fig. 3c also unambiguously identifies the coexistence of CeO_2_ and NiFe-MOFs (Fig. 3d). The dislocations are clearly observed in Fig. 3e, which indicates the existence of a defect-rich structure.

Subsequently, the research is focused on understanding intrinsic interactions of electronic charge transfer, and to then monitor how coordinated modification affects the optical properties of CeO_2_. The excitation spectra of CeO_2_-OA display intense bands in the ultra-violet region (Fig. 3f). As expected, the excitonic transition bands of CeO_2_-BDC were red shifted in comparison to CeO_2_-OA, and the band of 2D CeO_2_@NiFe-MOF heterostructures is up-shifted compared to that of CeO_2_-OA and CeO_2_-BDC, which could be ascribed to increased scattering effects arising from aggregate and electronic interactions between CeO_2_ and NiFe-MOFs.^42,43^ To further elucidate the influence of coordinated functionalization for optical properties of CeO_2_, UV-vis diffuse reflection spectroscopy was carried out, which showed that band gap energies are 3.03 eV and 2.92 eV for CeO_2_-OA and CeO_2_-BDC, respectively (Fig. 3g), indicating that surface coordination can result in fine-tuning of electronic and optical properties of CeO_2_. The red-shift of band gap energy can be also assigned to the increase of surface strain and defects from CeO_2_-OA to CeO_2_-BDC. Conversely, CeO_2_@NiFe-MOFs exhibit a broad band gap, which can be attributed to the increase of oxygenated groups and indicating the CeO_2_ surface coordinated with NiFe-MOFs. Different characteristics of CeO_2_ after coordination have been demonstrated. The Raman spectrum signal of CeO_2_-BDC (Fig. 3h) is asymmetrically broadened relative to that of pristine CeO_2_-OA, caused by the increase of the lattice constant.^44^ Meanwhile, the asymmetrical broadening also indicates the decreasing CeO_2_ size. The decreasing size proves that carboxylic acids properly dissolve surface Ce of CeO_2_ and coordinate with CeO_2_. The tight bonding between CeO_2_ nanosheets and NiFe-MOF layers is the linchpin. Thus, carboxylic acid groups strongly coordinate with cerium and the tails anchor on the metal ions. Besides, the π-conjugated structure in carboxylic acid groups also provides a platform to facilitate electron transfer between them in the process of catalytic conversion.

**Fig. 4:**
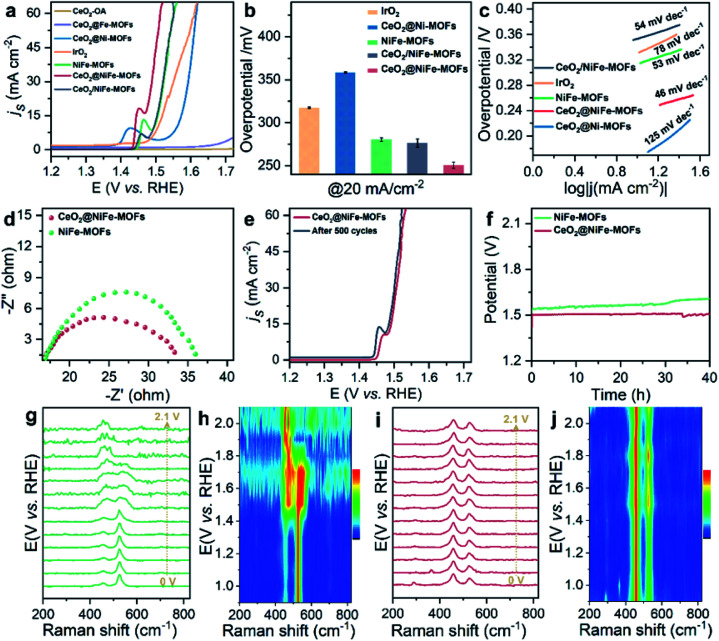
(a) OER polarization curves for different electrocatalysts in 1 M KOH solution. (b) The corresponding overpotentials to drive 20 mA cm^−2^. (c) Corresponding Tafel plots. (d) Nyquist plots of NiFe-MOFs and CeO_2_@NiFe-MOFs recorded at 1.53 V *versus* RHE. (e) Polarization curves of CeO_2_@NiFe-MOFs before and after 500 cycles. (f) Chronopotentiometry curves of the NiFe-MOFs and CeO_2_@NiFe-MOFs at 20 mA cm^−2^. *In situ* electrochemical Raman spectra of (g) NiFe-MOFs and (i) CeO_2_@NiFe-MOFs under different applied potentials. (h and j) Corresponding contour plots of NiFe-MOFs and CeO_2_@NiFe-MOFs.

The OER is chosen as the primary catalytic model to verify the catalytic performance because NiFe-MOFs have a good water oxidation potential, because Fe incorporation enhances the reducibility of Ni in NiFe-MOFs, thereby potentially increasing the generation of oxygen vacancies and facilitating OER catalytic activity.^45,46^ Here, we mainly emphasize the importance of coordination of ultrathin CeO_2_ in promoting catalytic performance. In comparison, control groups and commercial IrO_2_ catalysts toward the OER were tested under the same conditions of a standard three-electrode measuring system in 1 M KOH solution at room temperature. Control groups were also prepared (Fig. S18–S20†). As revealed by the linear sweep voltammetry (LSV) polarization curves with *iR* correction (Fig. 4a), CeO_2_-OA shows very limited OER activity, and CeO_2_@NiFe-MOFs are testified to have the best activity toward the OER with the lowest overpotential of 248 mV at a current density of 20 mA cm^−2^, and outperform control groups at the same current density. The detailed comparison of the overpotentials of these samples is presented in Fig. 4b, and the electrocatalytic activity follows this order: CeO_2_@NiFe-MOFs (248 mV) > CeO_2_/NiFe-MOFs (280 mV) > NiFe-MOFs (282 mV) > IrO_2_ (297 mV) > CeO_2_@Ni-MOFs (352 mV). In addition, OER catalytic activity of CeO_2_@NiFe-MOFs was also measured at various temperatures to investigate the impact of temperature on catalytic activity (Fig. S21†). According to the experimental results, electrocatalytic activity was positively correlated with temperature, implying feasible application of CeO_2_@NiFe-MOFs in alkaline solution at various temperatures. Tafel plots obtained from LSV curves were further used to evaluate the OER kinetics of the prepared samples (Fig. 4c). As expected, the Tafel slope of CeO_2_@NiFe-MOFs is lower than that of the other control groups, indicating that NiFe-MOFs with coordination of CeO_2_ shows better OER kinetics. Benefiting from the unique electronic configurations of cerium, the coordination of CeO_2_ may optimize the electronic structure and spatial arrangements of materials.^47^ In addition, NiFe-MOFs are nonuniformly distributed on the surface of CeO_2_ nanosheets, which can expose more active sites and enhance catalytic performance. Notably, the summary and comparison of CeO_2_@NiFe-MOFs and different materials are listed in Table S1.† Comparatively, CeO_2_@NiFe-MOF heterostructures in this work have relatively better performance and stability.

Electrochemical impedance spectroscopy (EIS) was carried out to investigate the charge transfer resistance (Fig. 4d) for CeO_2_@NiFe-MOFs, and the smaller semi-circular diameter of Nyquist plots indicated that the coordination of CeO_2_ effectively enhanced the electron transfer rate of NiFe-MOFs, implying that there was charge transfer between CeO_2_ and MOFs. Long-term electrocatalytic stability is an important challenge for electrocatalysts under alkaline conditions, and especially some unsupported MOF electrocatalysts are prone to degeneration.^4^ Therefore, it is essential to survey the stability to shed light on the importance of CeO_2_ coordination. As seen in Fig. 4e, the activity of CeO_2_@NiFe-MOFs shows almost no decay after 500 cycles. As shown in Fig. 4f, the stability of NiFe-MOFs and CeO_2_@NiFe-MOFs was evaluated by chronopotentiometric measurement at *J* = 20 mA cm^−2^. Obviously, the required potential of CeO_2_@NiFe-MOFs for 40 h shows almost no change, whereas the potential applied on NiFe-MOFs increases over 40 h. The morphology and structure of CeO_2_@NiFe-MOFs were again characterized after the stability test, and they were found to roughly preserve the initial morphology and structure (Fig. S22 and S23†). The above results reveal enhanced catalytic activity and structural durability of CeO_2_@NiFe-MOFs toward the OER. CeO_2_/NiFe-MOFs, which is an electrostatic composite material, show that CeO_2_ almost does not improve OER activity. Electrostatically associated materials have moderate binding strengths, and are unstable in solvents, while the coordinated linked conjugates have robust and strong bonding.^31,48^ Therefore, this further explains that the coordination of CeO_2_ can promote OER catalytic performance (Fig. S24–S26†).

To further explore electrochemical behavior of the obtained samples, *in situ* electrochemical Raman spectroscopy measurements were conducted to capture structural evolution under OER conditions. The potential-dependent spectral traces of samples were measured (1 M KOH). Fig. S27a† shows Raman spectra of the O–Ce–O vibration of CeO_2_-OA at approximately 446 cm^−1^ in electrolyte, which is down-shifted compared to that of dry CeO_2_-OA (457 cm^−1^, Fig. 3h) and the CeO_2_ fluorite crystal (460 cm^−1^).^49^ The Raman signals of CeO_2_-BDC emerge at approximately 217, 350, 440 and 462 cm^−1^ (Fig. S27c†), and the Raman signal at 217 cm^−1^ was previously ascribed to a functionalization related to phonon mode.^50^ The above results suggest lattice distortions and creation of a fluorite crystal unit of CeO_2_ indicating that CeO_2_-OA and CeO_2_-BDC present excellent stability with increasing applied potential. As for NiFe-MOFs, two main Raman peaks at 456 and 525 cm^−1^ are correlated with the bending and stretching vibration modes of Ni–O in NiOOH, respectively. NiOOH, acting as potential active sites in electrocatalysis, significantly decreases with increasing applied potential, and the Raman peak at 525 cm^−1^ disappears at 1.9 V *vs.* RHE (Fig. 4g and h). As expected, the intensity of Raman peaks centered at 458 and 524 cm^−1^, originating from CeO_2_@NiFe-MOFs, changes a little upon the potential increasing from 1.0 to 2.1 V *vs.* RHE (Fig. 4i and j) demonstrating that materials with CeO_2_ will give better mechanistic functionalities.

**Fig. 5:**
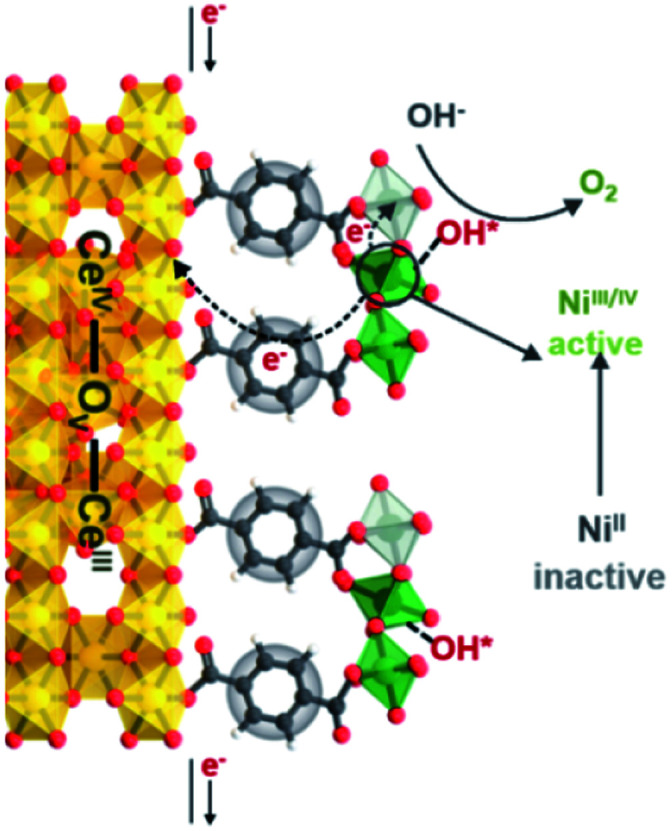
Schematic illustration of possible electron transfer paths occurring in the CeO_2_@NiFe-MOF heterostructures under electrocatalysis processes for the OER catalysis.

We propose the following electron transfer paths responsible for the observed enhancements in performance over CeO_2_@NiFe-MOF electrode under the OER conditions (Fig. 5). Monolayer NiFe-MOFs are coordinated to both the top and bottom surface of CeO_2_ nanosheets *via* joining carboxylic acid groups, and thus the π-conjugated structure in carboxylic acid groups will facilitate electron transfer from potential NiOOH active species to the CeO_2_ substrate. Therefore, generating Ni^III/IV^ active species under the process of electrocatalysis then extracting electrons from adsorbate OH* and accomplishing desorption of the formed O_2_ (Fig. S28†) enhance the OER process, which is testified by the clearly observed enhancement in performance (Fig. 4a). In addition, CeO_2_ easily undergoes reversible charge transfer between Ce^4+^ and Ce^3+^ oxidation stages, which is advantageous for facilitating OER catalysis. On the other hand, oxygen vacancies on CeO_2_ functioning as effective electron trappers can capture electrons, and this is beneficial for the extraction of electrons from adsorbate OH* and promoting the electrocatalytic reaction. According to previous reports, CeO_2_[111] has been recognized to present the most stable surface, and CeO_2_[110] can very easily generate oxygen vacancies.^51,52^ Nevertheless, CeO_2_ nanosheets present the most stable surface of CeO_2_[111] in CeO_2_@NiFe-MOFs (Fig. S10c†); meanwhile, no typical peak of defect-induced mode at around 592 cm^−1^ was observed in the Raman spectra (Fig. 3h and 4i), which was attributed to the fact that carboxylic acid functional groups can heal the oxygen vacancies of CeO_2_. The result indicates that intrinsic properties of CeO_2_ rather than oxygen vacancies contribute to catalytic activity.

**Fig. 6:**
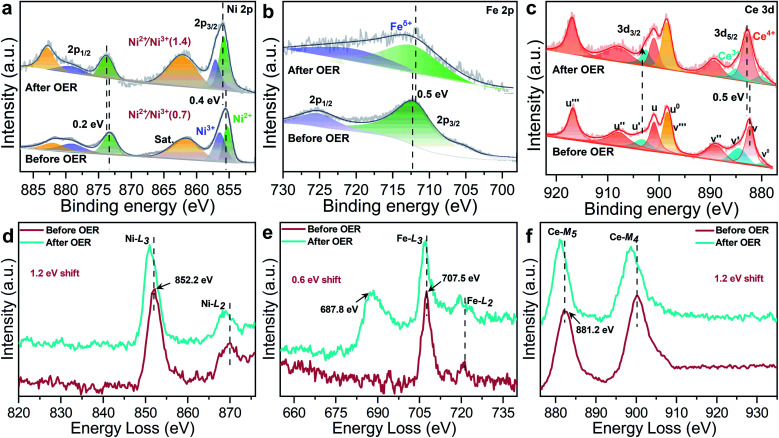
High-resolution XPS spectra of CeO_2_@NiFe-MOFs before and after the OER: (a) Ni 2p, (b) Fe 2p, and (c) Ce 3d. (d–f) The corresponding EELS spectra of CeO_2_@NiFe-MOFs before and after the OER: (d) Ni-L, (e) Fe-L, the peak at 687.8 eV is attributed to F-K (Fig. S31†), and (f) Ce-M.

The XPS spectra further confirm electronic behavior of CeO_2_@NiFe-MOFs before and after OER testing (Fig. 6a–c and S29†). Compared with the original CeO_2_@NiFe-MOFs, Ni 2p_3/2_ and 2p_1/2_ peaks of CeO_2_@NiFe-MOFs after OER show apparent shifts toward the higher binding energies by 0.4 eV and 0.2 eV, respectively (Fig. 6a) and Fe 2p peaks show shifts toward the lower binding energies by 0.5 eV (Fig. 6b), indicating the reduction of Fe to a lower valence state. This result suggests that there is partial electron transfer from Ni to Fe sites *via* the bridging O atoms.^38^ The intensity of the Ce^3+^ related peak at 903.8 eV also increases (Fig. 6c), demonstrating that the charge transfer occurs from NiOOH active species to the CeO_2_ substrate. In addition, the Ni^II^/Ni^III^ ratio on the surface of CeO_2_@NiFe-MOFs after OER increases, and likewise, the consistent reduction of the valence state is also observed from the negative shift in the corresponding electron energy loss spectra (Fig. 6d–f and S30†), indicating the reduction of the valence state to a lower valence state after OER testing. Therefore, the high valence state Ce^4+^ as electron-accepting sites on the pristine CeO_2_@NiFe-MOF surface significantly promotes intrinsic electron transfer from NiOOH to CeO_2_ through π-donation.^53^ Electron conjugation can improve electron communication between them,^54^ making a greater amount of coordinated NiOOH electrochemically active and then facilitating the generation of Ni^III/IV^ active species in the CeO_2_@NiFe-MOF electrode during catalysis.^43^ This could also be clearly evaluated by EIS analysis. Simultaneously, the oxygen storage capacity generated from the fast reversible variation between Ce^3+^ and Ce^4+^ and the surface defect-rich structure of ultrathin CeO_2_ serves as an oxygen buffer to enhance wettability and release stress from a drastic reaction.^55^ Though CeO_2_ is not a good OER catalyst, these results clearly demonstrate that CeO_2_ could essentially improve activity and stability of electrocatalysis by coordination with particular 2D MOF materials.^56^ In comparison with other uniformly structured counterparts, 2D heterostructures exhibit greater stability and provide more efficient charge transfer.^57,58^

The coordinated CeO_2_@NiFe-MOF heterostructures as a structural model were constructed, and their electrocatalytic performance was examined by using the OER as a model system. In the designed 2D heterostructures with a sandwich structure, CeO_2_ not only assisted the coordinately supported and confined MOFs to easily expose more accessible active sites, but also provided a surface defect-rich and unique electronic structure in favor of the catalytic reaction. Meanwhile, the organic–inorganic hybrid heterostructures provide sufficient surface area for the catalytic reaction. The structure and catalytic performance, as well as the key electron transfer in 2D heterostructures were well proved by the characterization studies. Therefore the clear coordination environment of the 2D heterostructures is beneficial to establish the catalytic process and elucidate the catalytic mechanism in the heterostructures. Besides, the clear coordinated combination is not only structurally suitable for 2D CeO_2_-supported MOFs, but also can be extended to a general strategy for the combination of 2D CeO_2_ and other molecular layers or precursors, where the interactions between metal oxides and molecular layer require a deeper understanding.^59^ As we developed this strategy to construct the integrated structure as a superior model system, researchers can be inspired to regulate other hybrid structures for the improvement of catalytic performance by considering the role of CeO_2_ coordination. Therefore, the hybrid materials of coordinated combination possess superiority as a model system, and high activity of 2D MOF-based or CeO_2_-based heterostructures can be expected by adjusting interactions between the constituents.

## Conclusions

In this work, by employing the means of coordination, we first construct ultrathin 2D CeO_2_@NiFe-MOF heterostructures. It proves the multiple effects of metal-coordinated interactions in CeO_2_@NiFe-MOF heterostructures. CeO_2_ nanosheets decorated with 2D NiFe-MOFs present different electronic and optical properties, as well as local strain. On the other hand, 2D NiFe-MOFs on the surface of the CeO_2_ nanosheet substrate not only present an excellent OER catalytic activity but also display good long-term stability, achieving a current density of 20 mA cm^−2^ at a low overpotential of 248 mV as well as long-term durability for at least 40 h. In addition, the ultrathin 2D CeO_2_ surface can also be functionalized by other organic ligands through this approach. As a consequence, an extensive class of multi-functional 2D CeO_2_ materials can be predictably synthesized by rationally tailoring structures of organic ligands. It is anticipated that our study will provide a reference for the improvement of the catalytic process by using coordinated heterostructures and open new avenues for the formation of novel 2D MOF composites/heterostructures or multi-functional 2D CeO_2_ for extensive applications.

## Data availability

All relevant data is presented in the paper and ESI.†

## Author contributions

Y. T. and L. L. contributed to valuable guidance and comments and supervised the project. H. A. performed the synthesis, characterizations and electrochemical experiments. Y. H. and Y. P. assisted with the HR-TEM testing and data analysis. The manuscript was written through contributions of all authors.

## Conflicts of interest

There are no conflicts to declare.

## Supplementary Material

SC-013-D2SC00308B-s001
