# Supplementary material for: Two-dimensional heterostructures built from ultrathin CeO2 nanosheet surface-coordinated and confined metal–organic frameworks with enhanced stability and catalytic performance
Source: Chem Sci. 2022 Feb 14;13(10):3035–44. doi: 10.1039/d2sc00308b (PMC8905825; doi:10.1039/d2sc00308b)
Supplement: SC-013-D2SC00308B-s001 [file SC-013-D2SC00308B-s001.pdf]

Electronic Supplementary Material (ESI) for Chemical Science.
This journal is © The Royal Society of Chemistry 2022

Supporting Information

Two-dimensional heterostructures built from ultrathin CeO_2 nanosheets surface-coordinated and confined metal-organic frameworks with enhanced stability and catalytic performance

Haiyan An,[a] Yang Hu,[b] Nan Song,[a] Tingliang Mu,[a] Shiqiang Bai,[a] Yong Peng,[b] Liangliang Liu*[a] and Yu Tang*[a]

[a] State Key Laboratory of Applied Organic Chemistry, Key Laboratory of Nonferrous Metal Chemistry and Resources Utilization of Gansu Province, College of Chemistry and Chemical Engineering, Lanzhou University, Lanzhou 730000, China

[b] Key Laboratory of Magnetism and Magnetic Materials of Ministry of Education, School of Physical Science and Technology, Lanzhou University, Lanzhou 730000, China

*Corresponding author, E-mail: liull@lzu.edu.cn; tangyu@lzu.edu.cn

1. Experimental Section

1.1 Chemicals

Ascorbic acid, cerium (III) acetate hydrate ($Ce(C_2H_3O_2)_3\cdot nH_2O$), nickel (II) chloride hexahydrate ($NiCl_2\cdot 6H_2O$), iron (II) chloride tetrahydrate ($FeCl_2\cdot 4H_2O$), terephthalic acid (1,4-BDC), *N, N*-dimethylformamide (DMF), *N, N*-dimethylacetamide (DMAC), oleic acid (OA), oleylamine (OAm), sodium hydroxide (NaOH), triethylamine (TEA), 1,2,4,5-Benzenetetracarboxylic acid (PMA), ethanol (C_2H_5OH, AR) and cyclohexane (C_6H_{12}, AR) were obtained from commercial suppliers and used without further purification.

1.2 Synthesis of ultrathin CeO_2- OA nanosheets.

Ce $(CH_3COO)_3\cdot nH_2O$ (634.5 mg) and ascorbic acid (176 mg) were directly added into a mixture of (1.58 mL) OA and OAm (4.94 mL) (solution B) in a three-necked flask (100 mL) at room temperature. The resulting slurry was heated to 120 °C with vigorous stirring under vacuum, at 15 minutes after the solution reaches 120 °C, the solution was quickly extracted with a 20 mL glass syringe and then injected into another three-necked flask with a solution of OA (1.9 mL) and OAm (5.92 mL) (solution A) under 310 °C and Ar atmosphere. At 30 minutes after injection, the solution was cooled down to about 120 °C under Ar atmosphere and the products were flocculated by adding 40 mL ethanol into the reaction mixture and centrifugated under 8000 rpm for 10 minutes. The product was collected and washed with ethanol (40 mL) and cyclohexane (5 mL) for another 3 times.

1.3 Synthesis of ultrathin CeO_2-BDC nanosheets.

Ligand Exchange: the process was performed under room atmosphere. In a typical ligand exchange of CeO_2-OA nanosheets with 1,4-H_2BDC, 50 mL of CeO_2-OA (50 mg) solution in hexane (1 mg/mL) was mixed with 50 mL of DMF containing 1,4-H_2BDC (25, 50, 100, 250 mg), respectively, noted as CeO_2-BDC (2/1), CeO_2-BDC (1/1), CeO_2-BDC (1/2) and CeO_2-BDC (1/5), respectively. The CeO_2 nanosheets gradually transferred from hexane to DMF under vigorous stirring to obtain CeO_2-BDC, respectively. The bottom phase was separated and rinsed with fresh DMF, finally, washed with ethanol and dried at 60 °C for 12 h.

1.4 Synthesis of ultrathin CeO_2-PMA and CeO_2-OH nanosheets.

The preparation process was same as that of CeO_2-BDC, except that 1,4-BDC was replaced by PMA and -OH, respectively.

1.5 Synthesis of CeO_2@NiFe-MOFs.

First, CeO_2-BDC (1/1) (40 mg) was dispersed into the DMAC (15 mL) under magnetic stirring, 0.4 mL TEA was quickly injected into the solution. Subsequently, the aqueous

solution of $NiCl_2 \cdot 6H_2O$ (24 mg) and $FeCl_2 \cdot 4H_2O$ (7 mg) in 15.0 mL ultrapure water were dropwise added to DMAC solution of CeO_2-BDC, the solution was stirred for 30 min to obtain a uniform suspension. Finally, the mixed solution was transferred into a 50 mL Teflon vessel at 150 °C for 3 h. After cooling to room temperature, the precipitate was collected via centrifugation, washed with DMAC and ethanol (3 ~ 5 times), and dried at 60 °C for 12 h.

1.6 Synthesis of other CeO_2@NiFe-MOFs.

The preparation process was same as that of CeO_2@NiFe-MOFs, except that CeO_2-BDC (1/1) were replaced by CeO_2-BDC (2/1), CeO_2-BDC (1/2) and CeO_2-BDC (1/5), the corresponding CeO_2@NiFe-MOFs denoted as CeO_2@NiFe-MOFs (2/1), CeO_2@NiFe-MOFs (1/2) and CeO_2@NiFe-MOFs (1/5), respectively.

1.7 Synthesis of NiFe-MOF nanosheets.

The preparation process was same as that of CeO_2@NiFe-MOFs, except that CeO_2-BDC (40 mg) was replaced by 1,4-H_2BDC (40 mg).

1.8 Synthesis of CeO_2@Ni-MOFs and CeO_2@Fe-MOFs.

The preparation process was same as that of CeO_2@NiFe-MOFs.

1.9 Characterizations

powder X-ray diffraction (PXRD), which was conducted on a Rigaku MiniFlex600 X-ray diffractometer using CuKa radiation. The surface images were recorded on a transmission electron microscopy (TEM, Tecnai G2Tf20) at 200 kV and 100 K. X-ray photoelectron spectra (XPS) spectra were recorded on a Shimadzu Axis Supra device and corrected using C1s line at 284.8 eV. Raman spectra of samples were measured with a Lab RAM HR Evolution (532 nm). The Brunauer-Emmett-Teller (BET) specific surface area of samples was determined using N_2 adsorption-desorption on an ASAP2020M. The FT-IR spectra were recorded by using Nicolet Nexus-670 FTIR spectrometer with KBr pellets technique. Thermogravimetric analyses (TGA) were performed on a Mettler Toledo Star System under a nitrogen atmosphere at a heating rate of 10 $°C \cdot min^{-1}$. The thickness of the nanosheets was measured by atomic force microscopy (AFM) using a Dimension-Icon (Bruker). UV-vis absorption and UV-vis diffuse reflection spectra were obtained on UV-2600. Electron energy loss spectroscopy (EELS) was recorded on Themis Z spherical aberration corrected transmission electron microscope Titan Cubed Themis G2 300.

2. Electrochemical Measurements

Electrochemical measurements were conducted in a typical three-electrode glass cell by an Electrochemical Workstation (CHI 760E). A saturated Hg/HgO electrode and Pt plate were used as reference electrode and counter electrode, respectively, the working electrode is a glassy carbon electrode coated uniformly with electrocatalysts. The potential was converted to reversible hydrogen electrode (RHE) via a Nernst equation: E (RHE) = E (Hg/HgO) + 0.059 × pH + 0.098 V. The overpotential (η) was calculated by η (V) = E (RHE) - 1.23 V. Cyclic voltammetry (CV) and Linear sweep voltammetry (LSV) measurements were recorded at a scan rate of 5 mV s^{-1} with the potentials between 0 V and 0.8 V vs. Hg/HgO in 1 M KOH solution. All polarization curves were calibrated with iR correction. Electrochemical impedance spectroscopy (EIS) was measured at 0.6 V vs. Hg/HgO with a frequency range from 100 KHz to 0.01 Hz. The electrochemical double-layer capacitance (C_{dl}) was measured by CV at a potential window of 0-0.1 V using the same working electrodes. CV curves were obtained at different scan rates of 10, 20, 30, 40, 50 mV s^{-1}. The current differences (ΔJ =Ja-Jc at the potential of 0.05 V) against scan rates were fitted to analyze electrochemically active surface area (ECSA). The durability was evaluated at a fixed galvanostatic current density of 20 mA cm^{-2}.

3. Supporting Figures

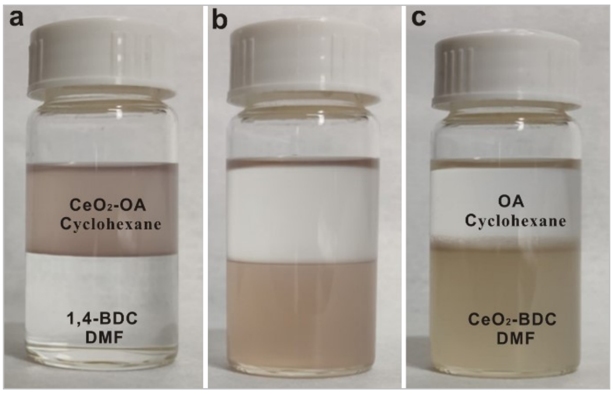

Fig. S1 Optical photographs of the (a) pristine CeO_2-OA, (b) CeO_2-BDC (after 24 hours) and (c) CeO_2-BDC (after 7 days) ligand exchange process in two phase solutions.

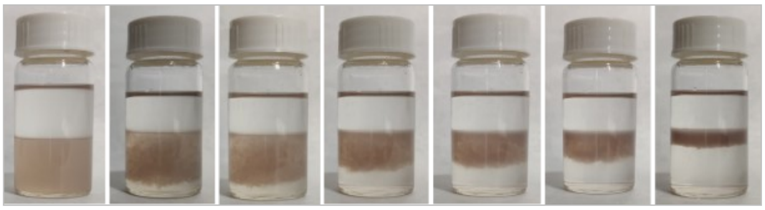

Fig. S2 Photograph phenomenon changes of the CeO_2-BDC (after 10 hours) under static condition.

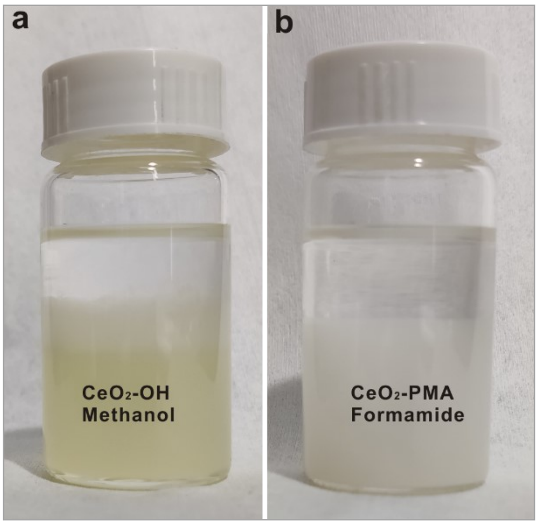

Fig. S3 Photographs of the (a) CeO_2-OH and (b) CeO_2-PMA (after 7 days) ligand exchange process in two phase solutions.

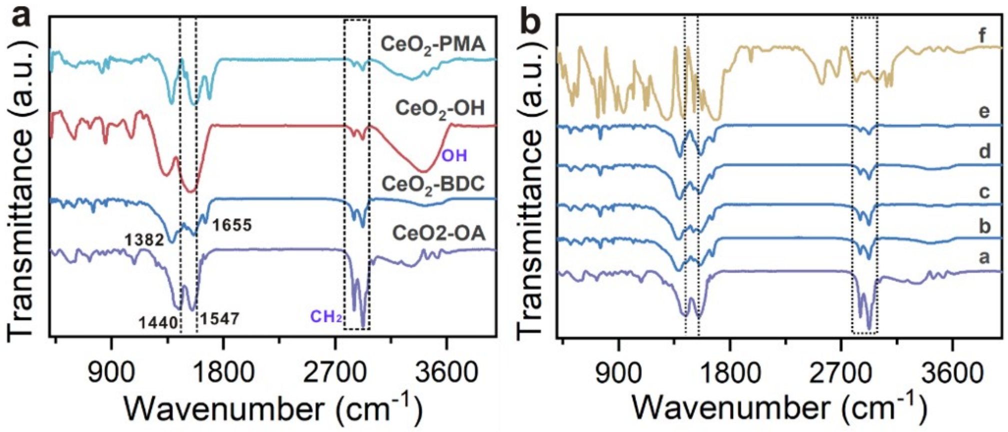

Fig. S4 The fourier transform infrared spectra (FT-IR) of (a) CeO_2-OA, CeO_2-BDC, CeO_2-OH and CeO_2-PMA. (b) FT-IR spectra of a: CeO_2-OA; b: CeO_2-BDC 2/1; c: CeO_2-BDC 1/1; d: CeO_2-BDC 1/2; e: CeO_2-BDC 1/5; f: 1,4-BDC.

FT-IR of CeO_2-OA nanosheets and CeO_2-BDC nanosheets revealed the degree of ligand exchange (Fig. S4a). The strong absorption peaks of C-H stretching vibrations (2700-3000 cm^{-1}) corresponding to the alkyl chain of OA are observed in the spectrum of CeO_2-OA.[1] We used 1,4-BDC, OH (hydroxyl) and PMA (1,2,4,5-benzenetetracarboxylic acid) to exchange OA respectively, the alkyl chain-related C-H stretching peaks with low intensity are still discernable in all of their FT-IR spectra, suggesting the incomplete replacement of organic ligands.[2] The vibrational intensity of C-H significantly decreases as the amounts of 1,4-BDC coordinating onto CeO_2 surface increases even there are incomplete ligand exchange (Fig. S4b). Since OA is a long chain molecule containing carboxylic acid, in the process of phase transfer, some OA molecules are strongly adsorbed on the surface of CeO_2 by carboxylic acid, and stabilize the structure of CeO_2. Therefore, the ligand exchange of oleic acid and short-chain oxygen-containing functional groups is not complete.

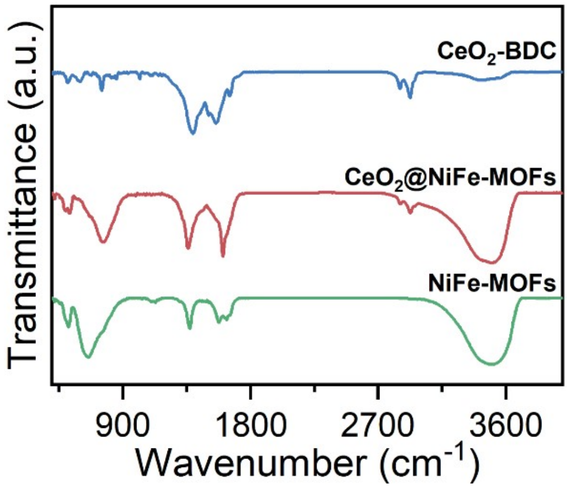

Fig. S5 FT-IR spectra of CeO_2-BDC, CeO_2@NiFe-MOFs and NiFe-MOFs.

The C-H peaks deriving from the incompletely ligand-exchanged CeO_2-BDC are observed in the FT-IR spectra of 2D CeO_2@NiFe-MOFs heterostructures, meanwhile, testifying the integration of CeO_2 and NiFe-MOFs nanosheets preliminarily.

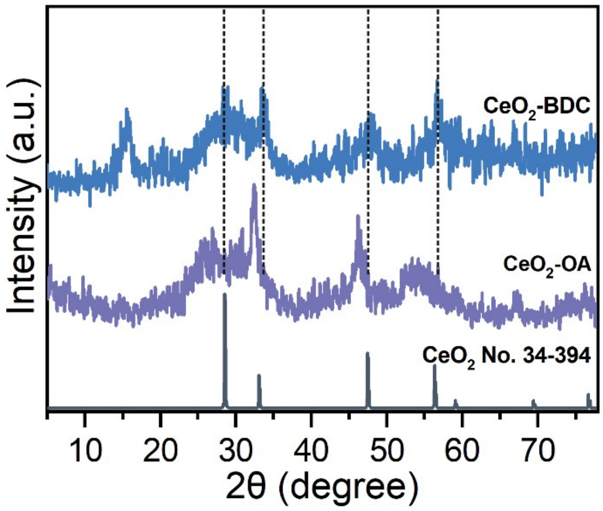

Fig. S6 PXRD patterns of the CeO_2-OA and CeO_2-BDC.

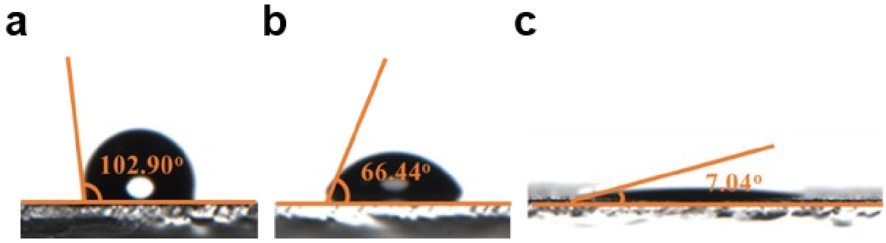

Fig. S7 Water contact angles of (a) CeO_2-OA, (b) CeO_2-BDC and (c) CeO_2@NiFe-MOFs.

Notably, CeO_2-OA possesses a superhydrophobic surfaces with a higher water contact angle of bubble (102.9°), attributing to the hydrophobicity of the alkyl chain of OA. As expected, CeO_2-BDC displays hydrophilic surfaces with a lower contact angle (66.4°), demonstrating the modification of 1,4-BDC. Meanwhile, CeO_2@NiFe-MOFs heterostructures is found to be significantly superhydrophilic (7.0°), the surface wetting property implies that the CeO_2@NiFe-MOFs is conducive to the electrolyte affinity and permeation, which is possibly benefit to the OER process.[3]

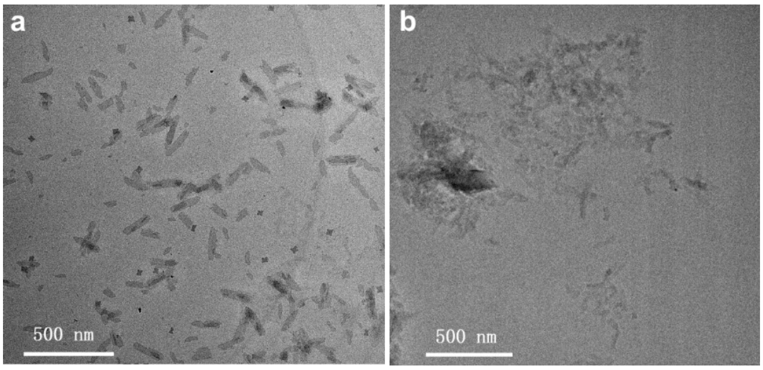

Fig. S8 The morphology of (a) CeO_2-OA and (b) CeO_2-BDC.

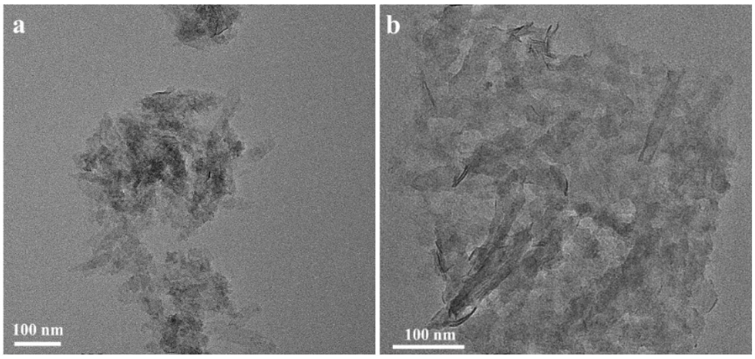

Fig. S9 The morphology of (a) CeO_2-OH and (b) CeO_2-PMA.

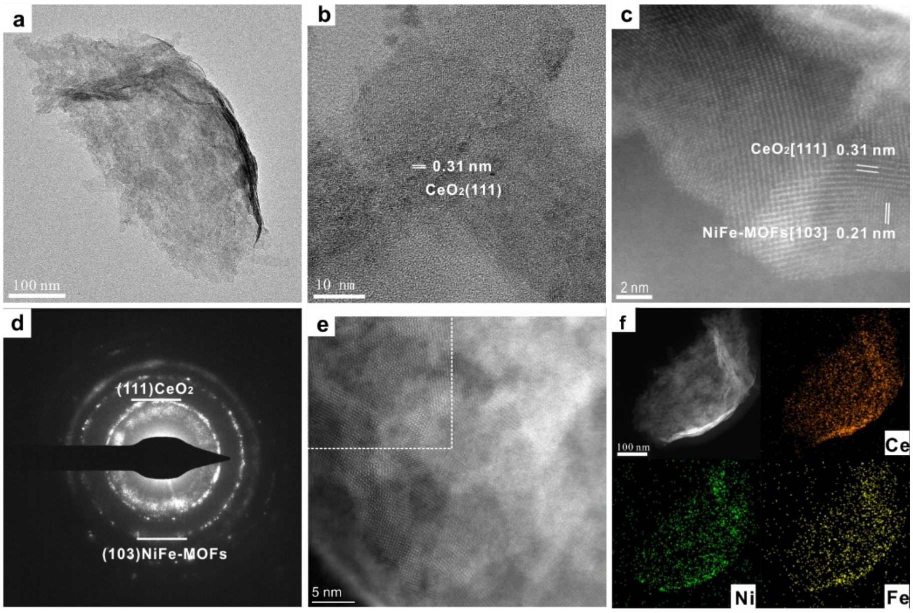

Fig. S10 (a) TEM image and (b-c and e) HR-TEM images of 2D CeO_2@NiFe-MOFs heterostructure. (d) The corresponding selected area electron diffraction (SAED) pattern of (a). (f) Corresponding elemental mapping images.

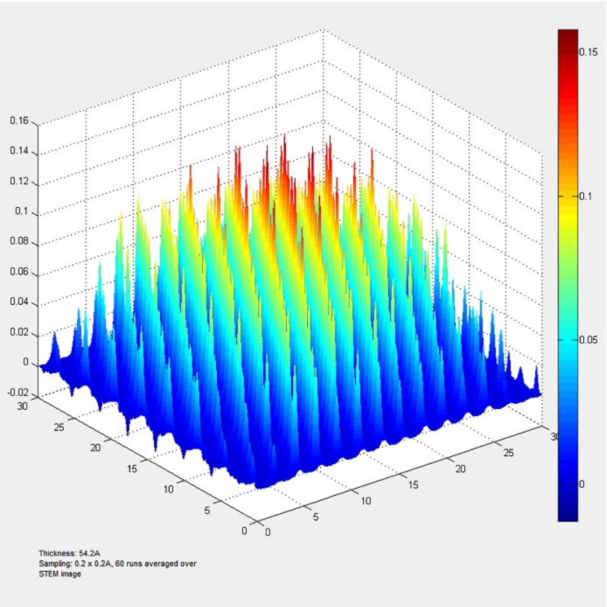

Fig. S11 Three-dimensional atom intensity profile of 2D CeO_2@NiFe-MOFs heterostructure.

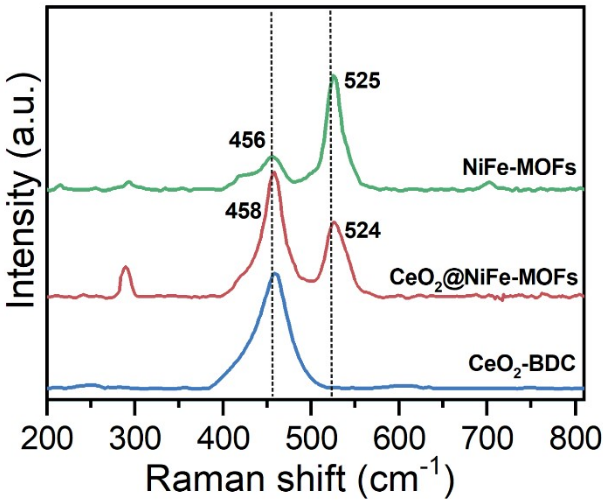

Fig. S12 Raman spectroscopy of CeO_2-BDC, NiFe-MOFs and CeO_2@NiFe-MOFs.

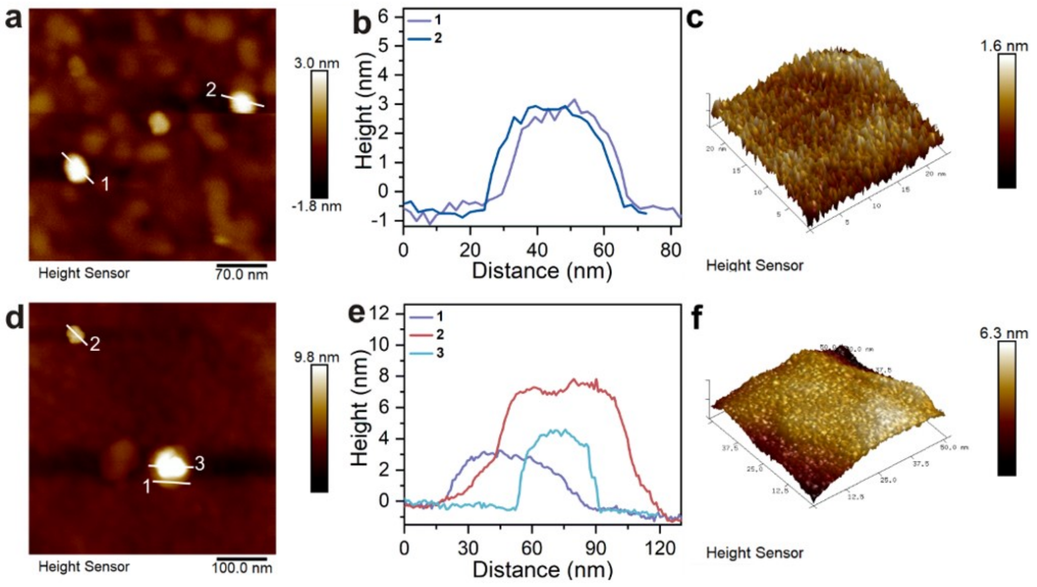

Fig. S13 AFM images of (a, c) CeO_2-OA and (d, f) CeO_2@NiFe-MOFs (b, e) the corresponding height profile.

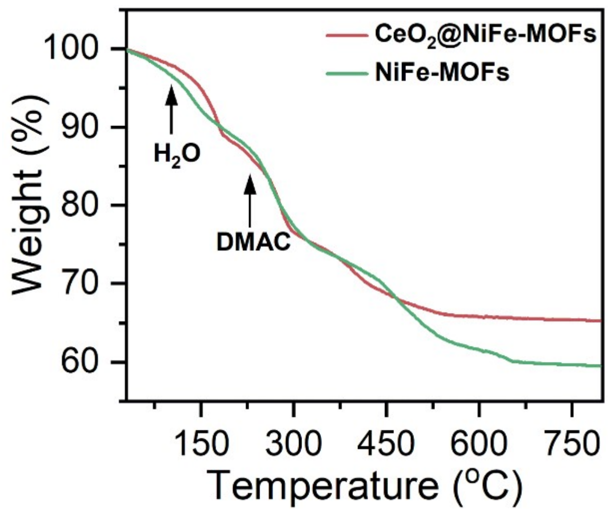

Fig. S14 Thermogravimetric analysis curves of CeO_2@NiFe-MOFs and NiFe-MOFs.

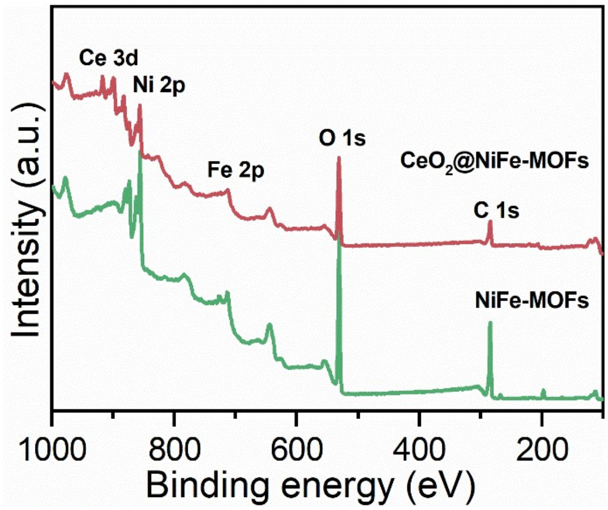

Fig. S15 XPS spectra of survey scan of CeO_2@NiFe-MOFs and NiFe-MOFs.

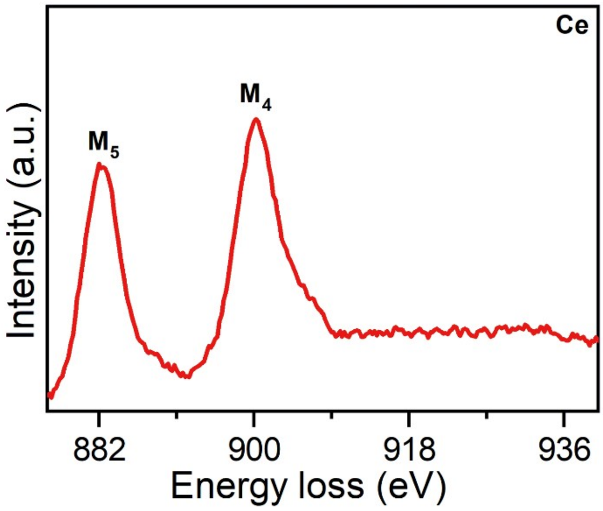

Fig. S16 The experimental EELS spectra of the Ce M-edge.

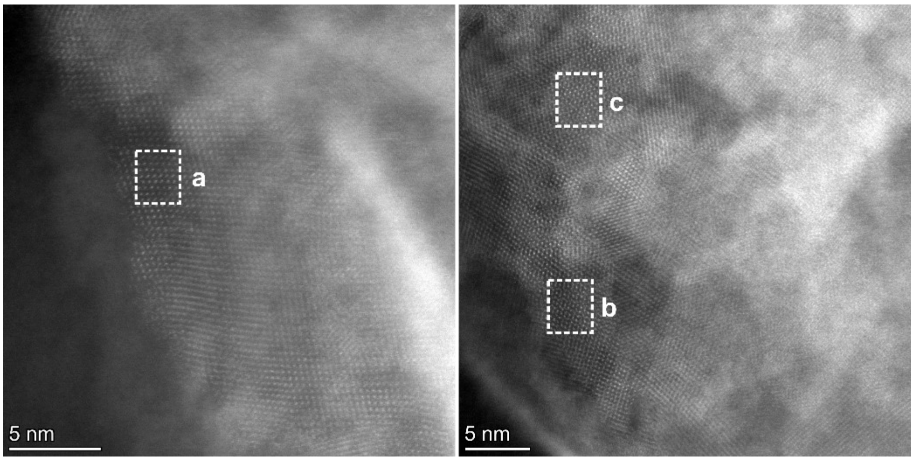

Fig. S17 HR-TEM images of CeO_2@NiFe-MOFs.

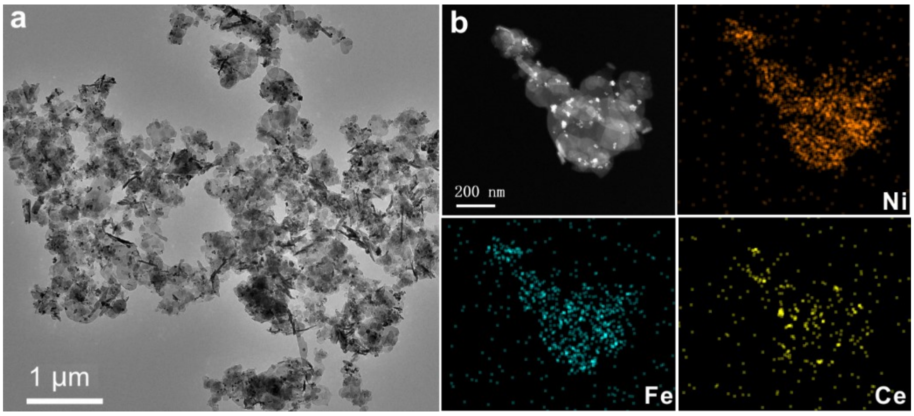

Fig. S18 (a) TEM image of the CeO_2/NiFe-MOFs. (b) HAADF-STEM image and elemental mapping images.

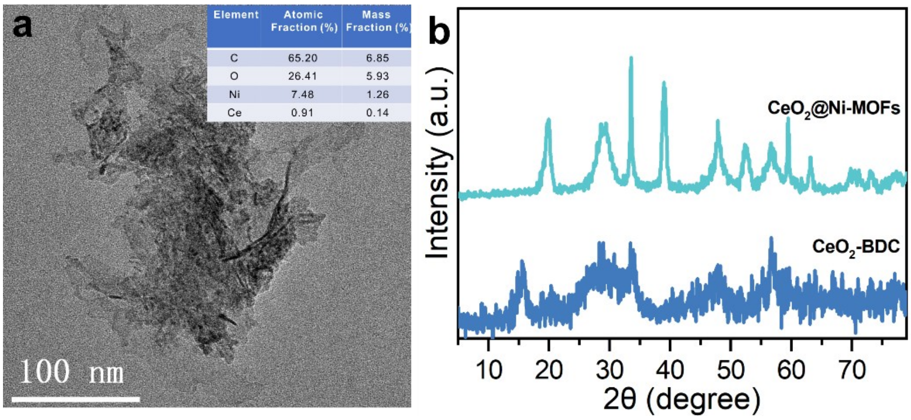

Fig. S19 (a) TEM image of the CeO_2@Ni-MOFs and EDS (insert). (b) PXRD.

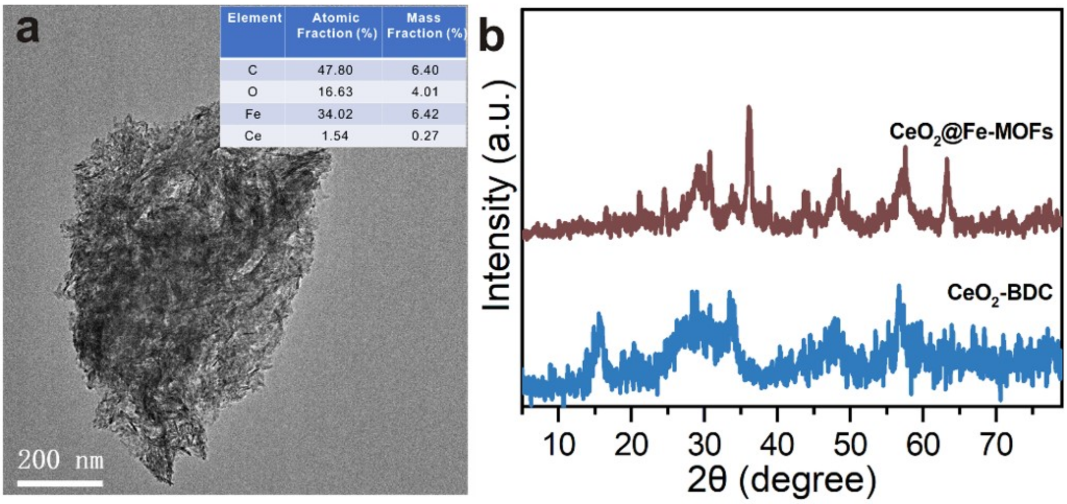

Fig. S20 (a) TEM image of the CeO_2@Fe-MOFs and EDS (insert). (b) PXRD.

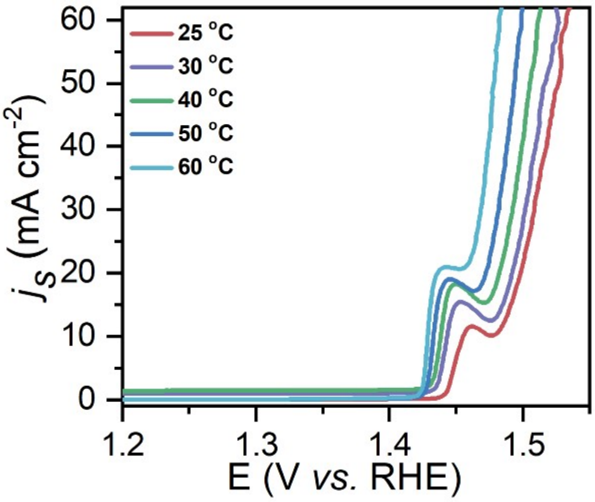

Fig. S21 Linear sweep voltammetry OER curves of CeO_2@NiFe-MOFs at different temperatures.

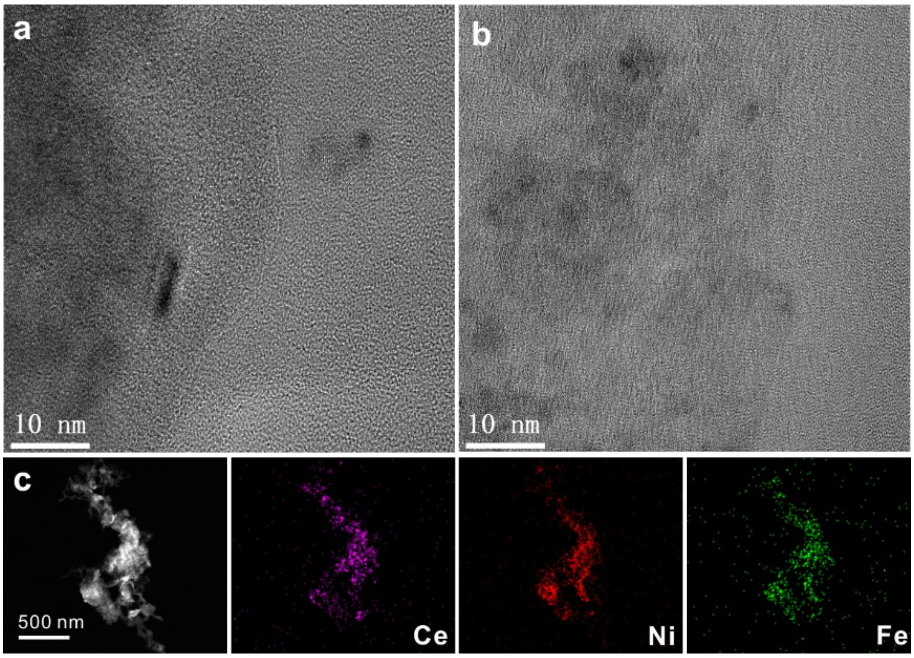

Fig. S22 (a, b) TEM images of CeO_2@NiFe-MOFs after 24 h chronopotentiometry OER tests at 20 mA cm^{-2}. (d) HAADF-STEM image and corresponding elemental mapping images.

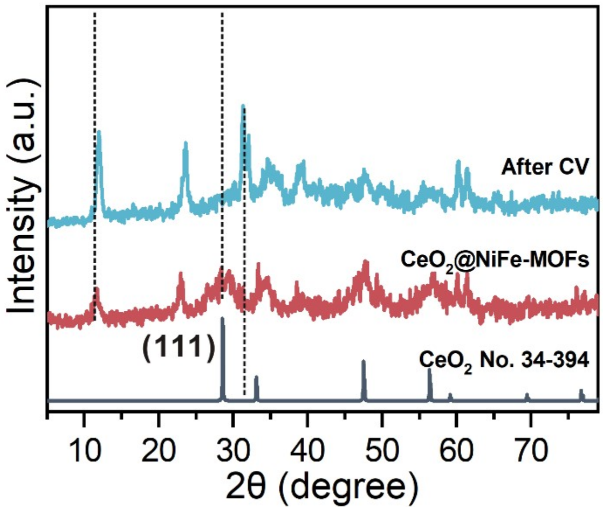

Fig. S23 PXRD patterns of the CeO_2@NiFe-MOFs after 24 h chronopotentiometry OER tests at 20 mA cm^{-2}.

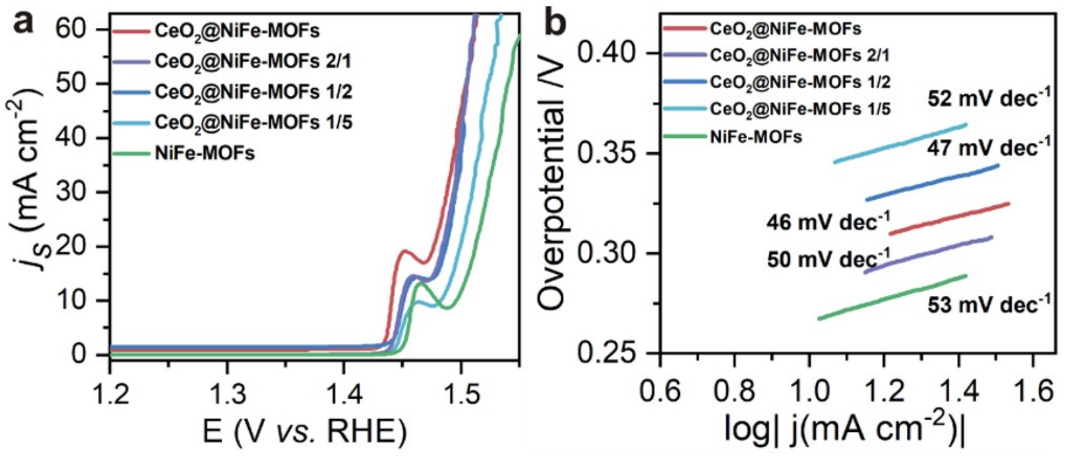

Fig. S24 (a) Linear sweep voltammetry OER curves of a series of CeO_2@NiFe-MOFs and (b) corresponding Tafel plots.

It is proved that the density of NiFe-MOFs could be tuned by varying feed ratios of CeO_2-OA and 1,4-BDC. On the other hand, it provides that the moderate coverage ratio of MOFs on CeO_2 surface can improve the catalytic performance. The loosened MOFs layer at the CeO_2 surface will remarkably increase open activity sites and allow faster diffusion of ions, which may be unstable. On the contrary, the MOFs are adhered to the CeO_2 surface in a compactness state may reduce activity sites and slow diffusion of ions.

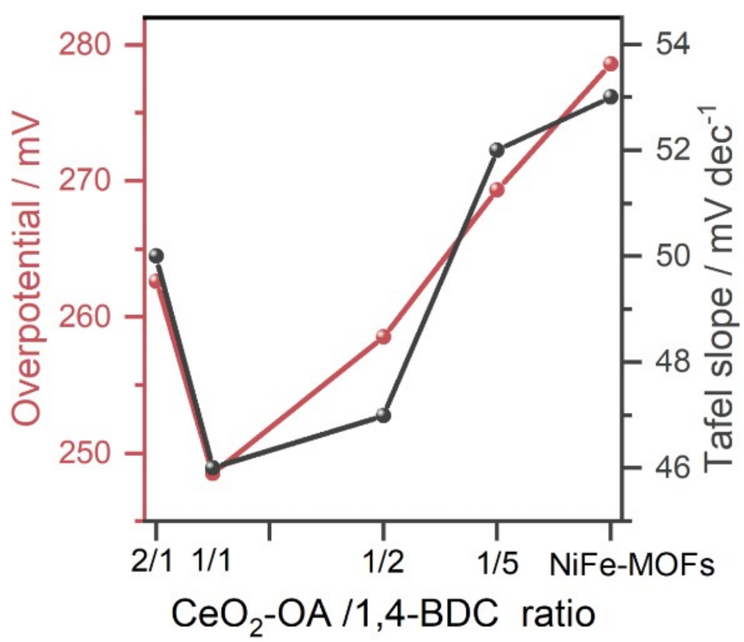

Fig. S25 The relationship between overpotential and Tafel plots of a series of CeO_2@NiFe-MOFs.

The overpotential and Tafel slope were plotted with mass ratio of CeO_2-OA and 1,4-BDC, the results exhibit that Tafel slope is consistent with change of overpotential.

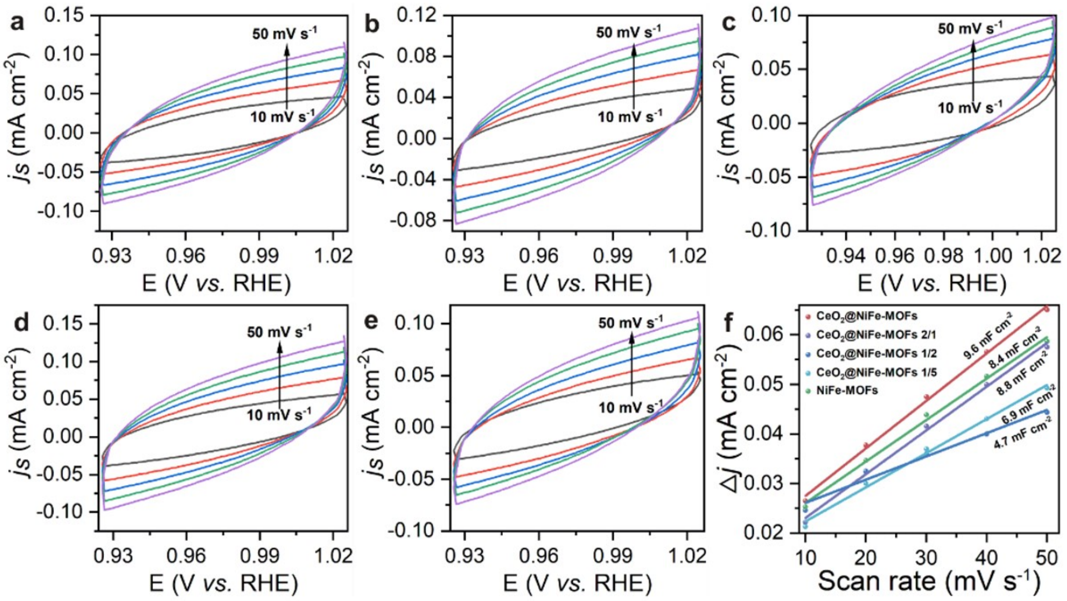

Fig. S26 CV curves in a potential range of 0.92-1.02 V versus RHE of (a) NiFe-MOFs, (b) CeO_2@NiFe-MOFs 2/1, (c) CeO_2@NiFe-MOFs 1/2, (d) CeO_2@NiFe-MOFs, (e) CeO_2@NiFe-MOFs 1/5 and (f) current density differences at 0.9754 V plotted against scan rate in a non-Faradaic range.

The plots of electrochemically active surface area (ECSA) were performed via CV measurement in non-Faradaic region, the double-layer capacitance (Cdl) was calculated by ΔJ = Ja-Jc at 0.9754 V vs. RHE versus scan rates, further indicating that coordination of CeO_2 can also affect number of catalytic active sites.

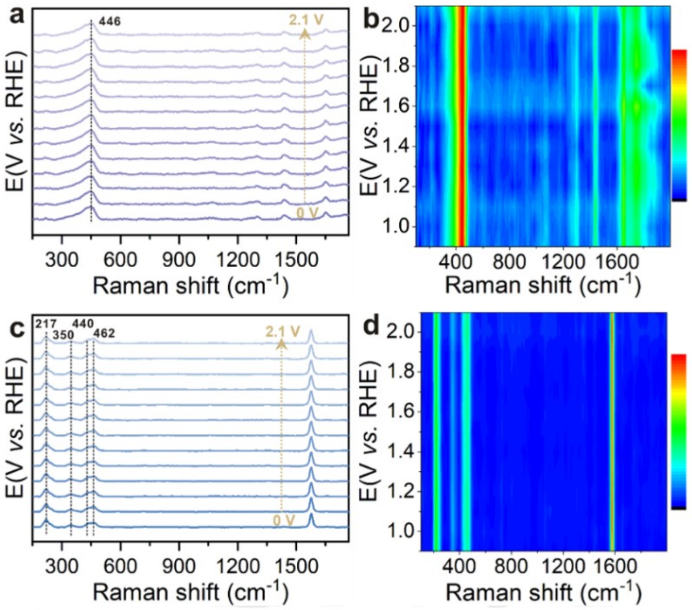

Fig. S27 In situ electrochemical-Raman spectra of (a) CeO_2-OA and (c) CeO_2-BDC under different applied potentials. (b and d) corresponding contour plots of CeO_2-OA and CeO_2-BDC.

$$OH^- \longrightarrow OH^* + e^- \quad (1)$$

$$OH^- + OH^* \longrightarrow H_2O + O^* + H^+ + e^- \quad (2)$$

$$OH^- + O^* \longrightarrow OOH^* + e^- \quad (3)$$

$$OH^- + OOH^* \longrightarrow O_2 + H_2O + e^- \quad (4)$$

Fig. S28 General OER reaction path in the alkaline electrolyte.

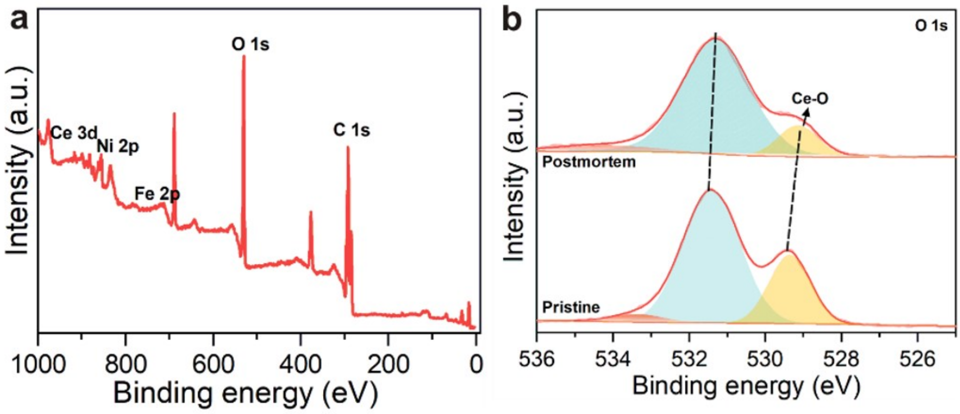

Fig. S29 XPS spectra of (a) survey scan of post-OER CeO_2@NiFe-MOFs and (b) O 1s.

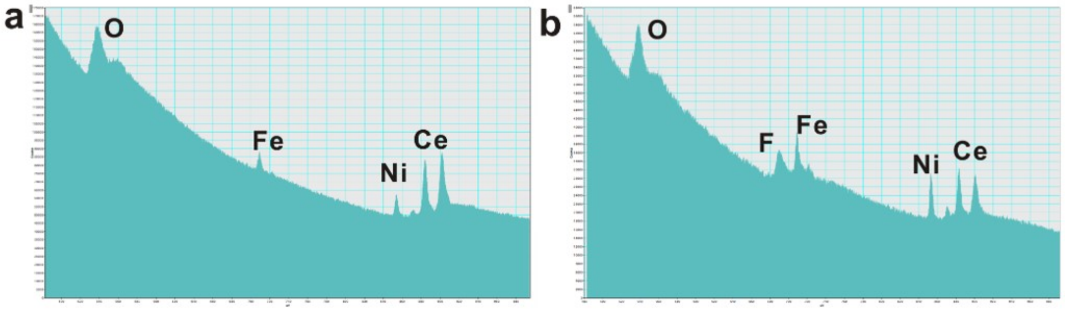

Fig. S30 EELS spectra of (a) CeO_2@NiFe-MOFs and (b) CeO_2@NiFe-MOFs after 24 h chronopotentiometry OER tests at 20 mA cm^{-2}.

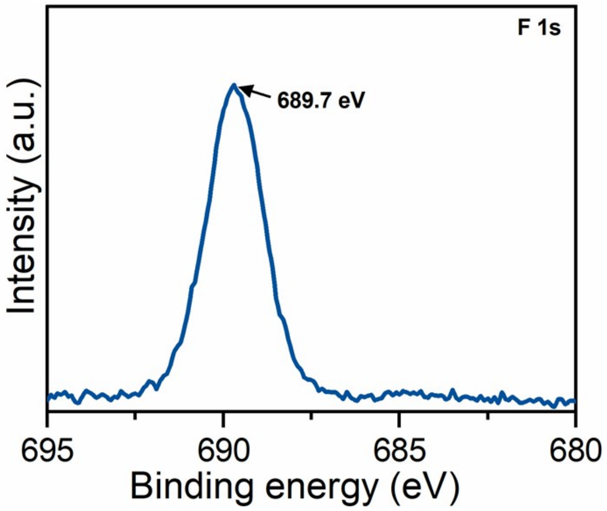

Fig. S31 XPS spectra of F 1s after 24 h chronopotentiometry OER tests at 20 mA cm^{-2}.

4. Supporting Table

Table. Comparison of electrocatalytic OER performance of CeO_2@NiFe-MOFs developed in current work with previous reported MOF electrocatalysts in the literatures.

Catalysts	Overpotential (mV)	Stability	Ref.
**CeO_2@NiFe-MOFs**	**248@20 mA cm^{-2}**	**40 h**	**Current work**
NiFe-UMNs	260@10 mA cm^{-2}	~3 h	[4]
NiFe-MOF/G	258@10 mA cm^{-2}	32 h	[5]
NiFe-MOF/Ni foam	240@10 mA cm^{-2}	~5 h	[6]
Ni-MOF@Fe-MOF	265@10 mA cm^{-2}	5 h	[7]
HG-NiFe	310@10 mA cm^{-2}		[8]
NiFe-TiO_2	346@10 mA cm^{-2}	5 h	[9]
NiCo@$NiCoO_2$-C	366 @20 mA cm^{-2}		[10]
NiOOH-h-CoO_2	350@10 mA cm^{-2}	12 h	[11]
Ni-BDC/$Ni(OH)_2$	320@10 mA cm^{-2}	20 h	[12]
FeNi@N-CNT	300@10 mA cm^{-2}	10 h	[13]
CoOOH-NS	253@10 mA cm^{-2}	100 h	[14]
M-CoO/CoFe LDHs	254@10 mA cm^{-2}	12 h	[15]
CeO_2/$Ni(OH)_2$/NOSCF	240@10 mA cm^{-2}		[16]
ZIF-9(III)/Co LDH-15	297@10 mA cm^{-2}	10 h	[17]
$NiCo_2S_4$@NiFe LDH	287@10 mA cm^{-2}	12 h	[18]
NiFe LDH/NiTe/NF	228@50 mA cm^{-2}	30 h	[19]
$Ti_3C_2T_x$-CoBDC	410@10 mA cm^{-2}	~3 h	[20]
Co-BPDC/Co-BDC-3	335@10 mA cm^{-2}	80 h	[21]

5. Supporting References

1. J. Huang, W. Liu, D. S. Dolzhnikov, L. Protesescu, M. V. Kovalenko, B. Koo, S. Chattopadhyay, E. V. Shenchenko and D. V. Talapin, *ACS Nano*, 2014, **8**, 9388-9402.
2. H. Jeong, S. Yoon, J. H. Kim, D.-H. Kwak, D. H. Gu, S. H. Heo, H. Kim, S. Park, H. W. Ban, J. Park, Z. Lee, J.-S. Lee, K. An and J. S. Son, *Chem. Mater.*, 2017, **29**, 10510-10517.
3. Q. Ma, P. Yin, M. Zhao, Z. Luo, Y. Huang, Q. He, Y. Yu, Z. Liu, Z. Hu, B. Chen and H. Zhang, *Adv. Mater.*, 2019, **31**, 1808249.
4. G. Hai, X. Jia, K. Zhang, X. Liu, Z. Wu and G. Wang, *Nano Energy.*, 2018, **44**, 345-352.
5. Y. Wang, B. Liu, X. Shen, H. Arandiyan, T. Zhao, Y. Li, M. Garbrecht, Z. Su, L. Han, A. Tricoli and C. Zhao, *Adv. Energy Mater.*, 2021, **11**, 2003759.
6. J. Duan, S. Chen and C. Zhao, *Nat. Commun.*, 2017, **8**, 15341.
7. K. Rui, G. Zhao, Y. Chen, Y. Lin, Q. Zhou, J. Chen, J. Zhu, W. Sun, W. Huang and S. X. Dou, *Adv. Funct. Mater.*, 2018, **28**, 1801554.
8. J. Wang, L. Gan, W. Zhang, Y. Peng, H. Yu, Q. Yan, X. Xia and X. Wang, *Sci. Adv.*, 2018, **4**, 7970.
9. R. Ramesh, S. Lee, S. Kim, J. Park, S. Lee, M. S. Kim, M. Baek, K. Yong, Y. Ye and J. Lee, *ChemElectroChem.*, 2018, **3**, 5130-5137.
10. H. Xu, Z.-X. Shi, Y.-X. Tong and G.-R. Li, *Adv. Mater.*, 2018, **30**, 1705442.
11. Z. Chen, L. Cai, X. Yang, C. Kronawitter, L. Guo, S. Shen and B. E. Koel, *ACS Catal.*, 2018, **8**, 1238-1247.
12. D. Zhu, J. Liu, L. Wang, Y. Du, Y. Zheng, K. Davey and S.-Z. Qiao, *Nanoscale*, 2019, **11**, 3599-3605.
13. Z. Tao, T. Wang, X. Wang, J. Zheng and X. Li, *ACS Appl. Mater. Interfaces.*, 2016, **8**, 35390-35397.
14. J. Zhou, Y. Wang, X. Su, S. Gu, R. Liu, Y. Huang, S. Yan, J. Li and S. Zhang, *Energy Environ. Sci.*, 2019, **12**, 739-746.
15. Z.-W. Gao, T. Ma, X.-M. Chen, H. Liu, L. Cui, S.-Z. Qiao, J. Yang and X.-W. Du, *Small*, 2018, **14**, 1800195.
16. Z. Liu, N. Li, H. Zhao, Y. Zhang, Y. Huang, Z. Yin and Y. Du, *Chem. Sci.*, 2017, **8**, 3211-3217.
17. W. Chen, C. Wang, S. Su, H. Wang and D. Cai, *Chem. Eng. J.*, 2021, **414**, 128784.
18. X. Feng, Q. Jiao, W. Chen, Y. Dang, Z. Dai, S. L. Suib, J. Zhang, Y. Zhao, H. Li and C. Feng, *Appl. Catal. B.*, 2021, **286**, 119869.
19. L. Hu, X. Zeng, X. Wei, H. Wang, Y. Wu, W. Gu, L. Shi and C. Zhu, *Appl. Catal. B.*, 2020, **273**, 119014.
20. L. Zhao, B. Dong, S. Li, L. Zhou, L. Lai, Z. Wang, S. Zhao, M. Han, K. Gao, M. Lu, X. Xie, B. Chen, Z. Liu, X. Wang, H. Zhang, H. Li, J. Liu, H. Zhang, X. Huang and W. Huang, *ACS Nano*, 2017, **11**, 5800-5807.
21. Q. Zha, F. Yuan, G. Qin and Y. Ni, *Inorg. Chem.*, 2020, **59**, 1295-1305.
